# Spectroscopic/Computational Characterization and the X-ray Structure of the Adduct of the V^IV^O–Picolinato Complex with RNase A

**DOI:** 10.1021/acs.inorgchem.1c02912

**Published:** 2021-11-30

**Authors:** Giarita Ferraro, Nicola Demitri, Luigi Vitale, Giuseppe Sciortino, Daniele Sanna, Valeria Ugone, Eugenio Garribba, Antonello Merlino

**Affiliations:** † Department of Chemical Sciences, University of Naples Federico II, I-80126 Napoli, Italy; ‡ Elettra−Sincrotrone Trieste, S.S. 14 km 163.5 in Area Science Park, 34149 Trieste, Italy; § Institute of Chemical Research of Catalonia (ICIQ), The Barcelona Institute of Science and Technology, 43007 Tarragona, Spain; ∥ Istituto di Chimica Biomolecolare, Consiglio Nazionale delle Ricerche, Trav. La Crucca 3, I-07100 Sassari, Italy; ⊥ Dipartimento di Scienze Mediche, Chirurgiche e Sperimentali, Università di Sassari, Viale San Pietro, I-07100 Sassari, Italy

## Abstract

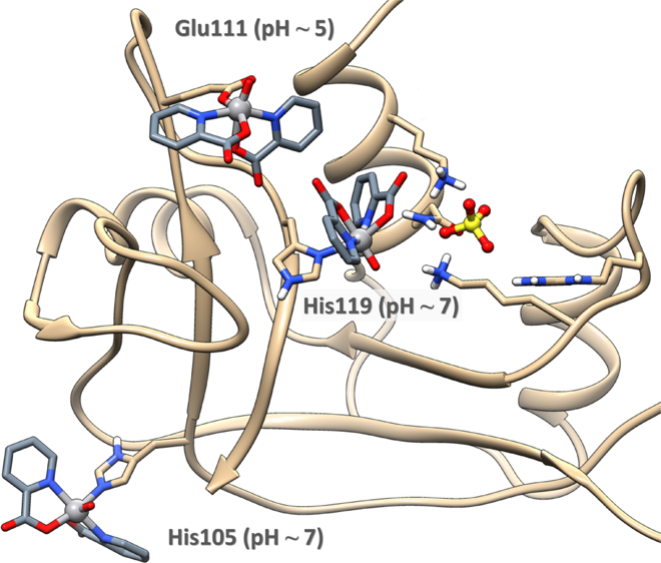

The structure, stability, and enzymatic activity of the adduct formed upon the reaction of the V–picolinato (pic) complex [V^IV^O(pic)_2_(H_2_O)], with an octahedral geometry and the water ligand in *cis* to the V=O group, with the bovine pancreatic ribonuclease (RNase A) were studied. While electrospray ionization-mass spectrometry, circular dichroism, and ultraviolet–visible absorption spectroscopy substantiate the interaction between the metal moiety and RNase A, electron paramagnetic resonance (EPR) allows us to determine that a carboxylate group, stemming from Asp or Glu residues, and imidazole nitrogen from His residues are involved in the V binding at acidic and physiological pH, respectively. Crystallographic data demonstrate that the V^IV^O(pic)_2_ moiety coordinates the side chain of Glu111 of RNase A, by substituting the equatorial water molecule at acidic pH. Computational methods confirm that Glu111 is the most affine residue and interacts favorably with the *OC*-6-23-Δ enantiomer establishing an extended network of hydrogen bonds and van der Waals stabilizations. By increasing the pH around neutrality, with the deprotonation of histidine side chains, the binding of the V complex to His105 and His119 could occur, with that to His105 which should be preferred when compared to that to the catalytically important His119. The binding of the V compound affects the enzymatic activity of RNase A, but it does not alter its overall structure and stability.

## Abstract 2

The structure, stability, and enzymatic activity of the adduct formed upon the reaction of [V^IV^O(picolinato)_2_(H_2_O)] with RNase A were studied through a combination of experimental and computational techniques, including X-ray crystallography. The binding of the V compound affects the enzymatic activity of RNase A, but it does not alter its overall structure and stability.

## Introduction

The development of vanadium complexes (VCs) in areas of catalysis, materials science, biology, and medicinal chemistry is a field of extensive research.^1^ VCs possess various biological roles in several living organisms, such as macroalgae, bacteria, and fungi. Ascidians,^2^*Polychaetes*,^3^ and mushrooms^4^ developed specific systems for uptake, transport, and storage of V. Moreover, in some of these organisms, V-dependent enzymes, like haloperoxidases or nitrogenases, were found.^1d,5^

**Scheme 1:**
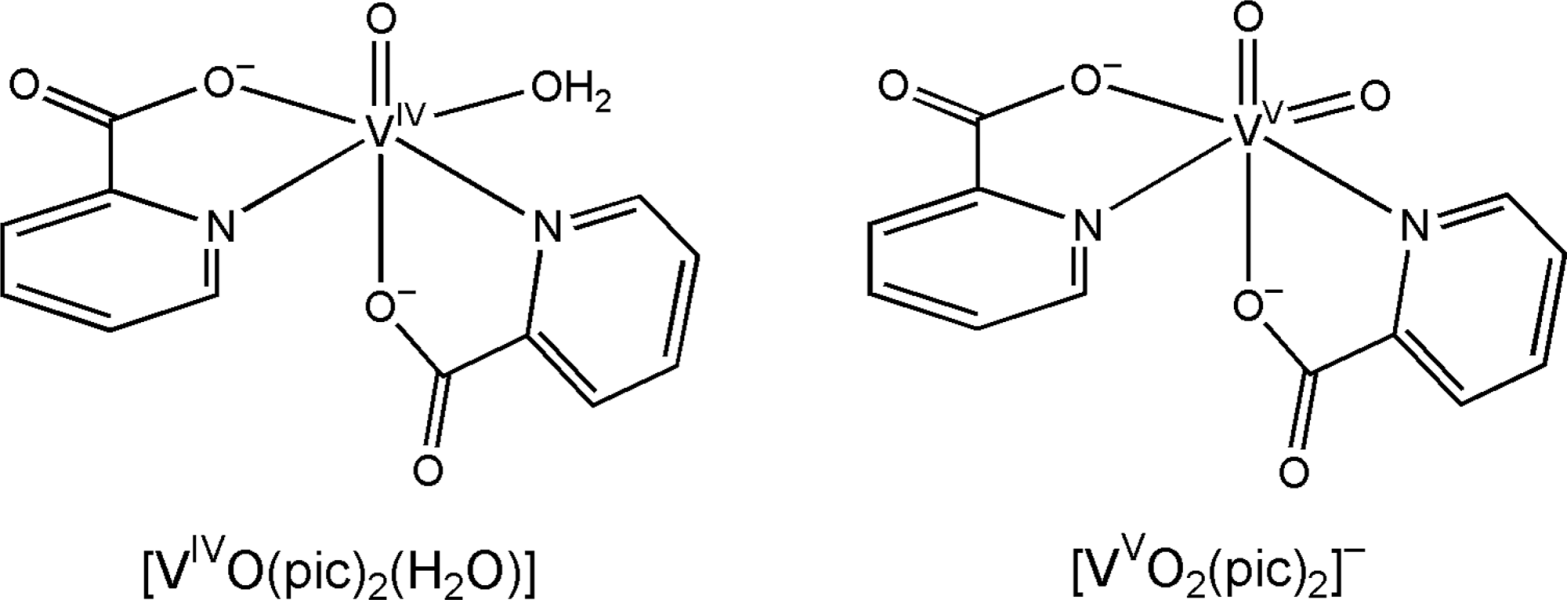
Structural Formulas of the V^IV^ and V^V^ bis-Chelated Complexes Formed by the Picolinato (pic) Ligand

VCs are suitable candidates in the search for novel anticancer agents,^6^ insulin enhancers,^1c,7^ and antibacterial^8^ and antiparasitic agents.^9^ Among several VCs exhibiting insulin-enhancing properties, species with the stoichiometry V^IV^O(carrier)_2_/V^V^O_2_(carrier)_2_ with carrier = maltolato (ma), 1,2-dimethyl-3-hydroxy-4(1*H*)-pyridinonato (dhp), picolinato (pic), and dipicolinato (dipic) have been extensively studied.^6e,6f,6h,6i^ V^IV^ and V^V^ complexes with the picolinato ligand and its derivatives are very promising potential drugs (Scheme 1). [V^IV^O(pic)_2_(H_2_O)] and [V^V^O_2_(picFF/picCN/picOH)_2_]^−^, where picFF, picCN, and picOH are 3,5-difluoropicolinato, 5-cyanopicolinato, and 3-hydroxypicolinato, are strong inhibitors of fatty acid mobilization and effective in the treatment of rats affected by diabetes induced with streptozotocin.^10,11^

The mechanism of action of these compounds is not entirely defined, but a crucial role in the pharmacokinetics of these molecules is played by their interaction with proteins.^5a,12,13^ For example, the antidiabetic activity of VCs is based on the inhibition of phosphatases by the H_2_V^V^O_4_^–^/V^IV^O(OH)_3_^–^ anion,^5a,6f,14,15^ and the interaction with human serum transferrin (HTf)^16,17^ and human serum albumin (HSA)^18,19^ in the serum and hemoglobin (Hb)^20,21^ inside the red blood cells plays a key role in the transport of vanadium species in the bloodstream. The involvement of HTf in the cellular uptake of VCs has also been proposed and discussed.^17,22,23^

Despite the high relevance of proteins as biomolecular targets for VCs, experimental data describing the X-ray structures of adducts of potential V drugs with proteins are still scarce. For V^IV^O(carrier)_2_, there is only one example in the literature: the adduct formed upon the reaction of [V^IV^O(pic)_2_(H_2_O)] with the hen egg white lysozyme (HEWL).^24^ In this structure, the V complex is covalently bound to the carboxylate group of the side chain of Asp52, which replaces the equatorial water molecule coordinated to the metal.^24^ However, from that paper of 2014, no other structures based on the V–carrier moiety were deposited in the Protein Data Bank (PDB).^25^

Recently, important developments in theoretical techniques and their applications were achieved, which allowed prediction of the covalent and noncovalent binding of a metal moiety to a protein.^26−28^ This approach has been also applied to VCs–protein systems, often combined with spectrometric and spectroscopic methods.^29^ In particular, for V^IV^O(carrier)_2_ compounds, electrospray ionization-mass spectrometry (ESI-MS) allows the determination of the number of moieties (V^IV^O^2+^, V^IV^OL^+^, or V^IV^OL_2_) bound to proteins, electron paramagnetic resonance (EPR) allows the definition of the type of amino acidic residue involved in the coordination of V^IV^O^2+^/V^IV^OL^+^/V^IV^OL_2_ fragments and computational techniques allow the prediction of the specific residues which interact with the metal ion as well as the stabilization of the adducts through secondary interactions such as hydrogen bonds (H-bonds) and van der Waals (vdW) contacts.^29^ The approach was applied to several proteins, such as the HEWL,^30−32^ myoglobin (Mb),^33^ ubiquitin (Ub),^31,32,34^ and cytochrome *c* (Cyt),^12f^ and used to rationalize the spectroscopic data in the literature on blood proteins and enzymes, such as HTf,^12d^ HSA,^19^ Hb,^12d^ immunoglobulin G (IgG),^12d^ vanadium bromoperoxidase (VBrPO),^12d^ and V^IV^O^2+^-substituted imidazoleglycerolphosphatase dehydratase (IGPD).^12d^ The results suggested that the residues involved in the covalent binding are mainly histidine (His), through the imidazole nitrogen, and aspartate (Asp) or glutamate (Glu), through the carboxylate group. The noncovalent binding is possible through exposed residues, mainly arginine (Arg), asparagine (Asn), glutamine (Gln), threonine (Thr), tryptophan (Trp), glutamate, and aspartate. However, despite these great progresses in predicting metal species binding on protein surfaces using theoretical methods, X-ray diffraction (XRD) structures are highly desirable to provide a complete atomic-level representation of metal–protein adducts.

Here, we studied the interaction of [V^IV^O(pic)_2_(H_2_O)] with bovine pancreatic ribonuclease (RNase A). RNases are members of a family of important enzymes that catalyze the scission of RNA into smaller molecules.^35^ RNase A is a prototype of this family.^36^ It has been recently pointed out that several therapies based on small molecules or oligonucleotides present the general aim of targeting RNA biology.^37^ RNase A has been also frequently used as a model protein in metalation studies, and structures with several metallodrugs,^38,39^ including cisplatin, carboplatin, oxaliplatin,^40^ and Ru-,^41^ Rh-,^42^ Pd-,^43^ Ir-,^44^ and Au-based drugs,^45^ are known. Recently, the metalation of arsenoplatin-1, a dual pharmacophore anticancer agent, has been presented.^46^ The adduct formed upon the reaction of RNase A with [V^IV^O(pic)_2_(H_2_O)] was characterized by a multiscale approach based on the combination of spectrometric/spectroscopic (ESI-MS, EPR, CD, and UV–vis), crystallographic (XRD), and computational methods (docking and QM/MM). Finally, ribonucleolytic activity assays were carried out.

## Experimental and Computational Section

### Chemicals

Water was deionized prior to use through the purification system Millipore Milli-Q Academic or purchased from Sigma-Aldrich (LC–MS grade). V^IV^OSO_4_·3H_2_O, 2-pyridinecarboxylic or picolinic acid, 1-methylimidazole (MeIm), ammonium acetate (NH_4_AcO), 4-(2-hydroxyethyl)piperazine-1-ethanesulfonic acid (HEPES), sodium citrate, and sodium acetate were Sigma-Aldrich products of the highest grade available and used as received. RNase A was purchased from Sigma-Aldrich (type XIIA). The neutral solid compound [V^IV^O(pic)_2_(H_2_O)] was synthesized according to the methods in the literature;^47^ among the four possible isomers, those with two nitrogen donors in the equatorial plane (*OC*-6-23 and *OC*-6-24, Scheme S1 of the Supporting Information) are favored and are in equilibrium in aqueous solution.^30,47,48^

### ESI-MS Measurements

The solutions for ESI-MS measurements were prepared by dissolving the solid complex [V^IV^O(pic)_2_(H_2_O)] in water or NH_4_AcO buffer solution (20 mM, pH 6.4) to obtain a V concentration of 1 mM. Subsequently, all the solutions were diluted in water, and an aliquot of a stock RNase A solution (500 μM in water or in 20 mM NH_4_AcO) was added to obtain a [V^IV^O(pic)_2_(H_2_O)]/RNase A ratio from 3/1 to 5/1 and a protein concentration of 5 μM. The final pH was 5.4 in water and 6.4 in buffer. The buffer concentration in the final samples was 1 mM. Argon was bubbled during all the operations to avoid V^IV^O^2+^ oxidation. ESI-MS spectra were recorded immediately after the preparation of the solutions.

Mass spectra in the positive-ion mode (ESI-MS(+)) were obtained on a Q Exactive Plus Hybrid Quadrupole-Orbitrap (Thermo Fisher Scientific) mass spectrometer. The solutions were infused at a flow rate of 5.00 μL/min into the ESI chamber. The spectra were recorded in the *m*/*z* range 200–3000 at a resolution of 140 000 and accumulated for at least 5 min to increase the signal-to-noise ratio. The instrumental conditions used for the measurements were the following: spray voltage, 2300 V; capillary temperature, 250 °C; sheath gas, 10 (arbitrary units); auxiliary gas, 3 (arbitrary units); sweep gas, 0 (arbitrary units); probe heater temperature, 50 °C. ESI-MS spectra were analyzed by using Thermo Xcalibur 3.0.63 software (Thermo Fisher Scientific), and the average deconvoluted monoisotopic masses were obtained through UniDec 4.4.0 software;^49^ the deviation from the expected masses was 2–3 Da.

### UV–Vis Electronic Absorption Spectroscopy and Circular Dichroism

Circular dichroism (CD) spectra were recorded with a Jasco J-715 spectropolarimeter equipped with a Peltier temperature controller. Far-UV measurements were carried out at a protein concentration of 14.6 μM in 10 mM sodium citrate buffer at pH 5.1 and at 20 °C, using a cell with an optical path length of 0.1 cm. Spectra, registered with a 50 nm min^–1^ scanning speed, a 2 s response time, a 1.0 nm data pitch, and a 2.0 nm bandwidth, were obtained averaging three scans.

Thermal unfolding curves were obtained by following the CD signal at 222 nm as a function of temperature in the 20–95 °C range, at a heating rate of 1.0 °C min^–1^. The denaturation temperatures (*T*_d_) were determined through analysis of the first derivative of the unfolding curves.

UV–vis spectra of [V^IV^O(pic)_2_(H_2_O)] were recorded on a Varian Cary 5000 UV–vis–NIR using the following parameters: wavelength range, 240–700 nm; data pitch, 1.0 nm; scanning speed, 400 nm/min; quartz cuvette path length, 1 cm; compound concentration, 50 μM. The spectra were collected at room temperature over time, monitoring the signal up to seven days, in the following experimental conditions: 10 mM sodium citrate buffer at pH 5.1, in the presence and in the absence of RNase A, at a protein-to-metal molar ratio of 1/3, and metal compound concentration of 150 μM.

### EPR Measurements

The solutions for EPR measurements were prepared by dissolving [V^IV^O(pic)_2_(H_2_O)] and RNase A in ultrapure water in order to get a V^IV^O^2+^ concentration of 0.8 mM and [V^IV^O(pic)_2_(H_2_O)]/RNase A molar ratios ranging from 3/1 to 1/3. Argon was bubbled through the solutions to ensure the absence of oxygen. HEPES buffer (0.1 M) was added to the solutions. The pH was adjusted to the desired value with diluted solutions of NaOH or H_2_SO_4_. Under these experimental conditions, the oxidation of V^IV^ to V^V^ is negligible, as proven by the examination of the relative intensity of the EPR signal as a function of the time.

The spectra of the model system [V^IV^O(pic)_2_(H_2_O)]/MeIm^16^ were examined to get information on the binding of the V^IV^O species to RNase A.

EPR spectra were recorded at 120 or 298 K with an X-band Bruker EMX spectrometer equipped with an HP 53150A microwave frequency counter. The microwave frequency was in the range of 9.40–9.41 GHz at 120 K and 9.84–9.85 GHz at 298 K; other instrumental settings (microwave power, modulation frequency, modulation amplitude, time constant, sweep time, and resolution points) are given in the Supporting Information. The spectra were immediately measured after the samples were transferred into the EPR tubes (120 K) or in a Bruker AquaX cell (298 K). Signal averaging was used to increase the signal-to-noise ratio;^50^ in particular, the number of scans for the high-field region of the spectra was 5 or 10. In the text, only the high-field part of the EPR signal is shown because it is more sensitive than the low-field region to the identity of the equatorial donors and the amount of several species in solution, as pointed out by some authors;^51^ the full spectra are reported in the Supporting Information.

To extract the spin Hamiltonian parameters, the spectra were simulated with the software WINEPR SimFonia.^52^

### Crystallization, X-ray Diffraction Data Collection, Structure Solution, and Refinement

RNase A (1.4 mM) was crystallized using the hanging drop vapor diffusion method and 22% PEG4K and 10 mM sodium citrate buffer at pH 5.1 as a reservoir at 20 °C. Crystals of the adduct were obtained by soaking metal-free protein crystals in a solution containing [V^IV^O(pic)_2_(H_2_O)] (saturated), 10 mM sodium citrate buffer at pH 5.1, and 22% PEG4K.

Diffraction data were registered at 100 K at the XRD2 beamline of the Elettra Synchrotron, Trieste, Italy.^53^ Crystals were cryoprotected using a solution of the reservoir with 25% glycerol. Data were indexed, integrated, and scaled using Autoproc.^54^ Data collection statistics are reported in Table S1. The phase problem was solved by molecular replacement using the RNase A structure deposited in the PDB under the accession code 1JVT (chain A)^55^ as the starting model. Several rounds of restrained individual atomic displacement parameter refinement, energy minimization, and individual *B*-factor refinement were carried out using Refmac;^56^ refinement cycles were followed by manual intervention based on observation of the electron density map carried out using Coot.^57^ Refinement statistics are reported in Table S1. The V atom position was identified by analysis of the anomalous difference electron density map. Model geometry was validated using the PDB validation server. Figures with electron density maps were drawn with UCSF Chimera software.^58^ The X-ray structure of the adduct [V^IV^O(pic)_2_]–RNase A was deposited in the PDB^25^ under the accession code 7P8R.

### Quantum and Docking Calculations

All the DFT calculations were carried out with Gaussian 16 (revision B.01).^59^ The V^IV^O complexes’ geometries and harmonic frequencies were computed at the level of theory B3P86/6-311g(d,p) using the SMD model^60^ for water. This guarantees a good degree of accuracy in the structure optimization of first-row transition metal complexes^61^ and, particularly, of VCs.^62^

Docking calculations were carried out through GOLD 5.8 software^63^ on the X-ray structure available in the Protein Data Bank (PDB) of free RNase A (PDB code: 1JVT(55)). The PDB structure was cleaned removing all the small molecules and crystallographic waters, and hydrogen atoms were added with the UCSF Chimera program.^58^ The protonation state of the amino acid side chains was computed by means of the PROPKA algorithm.^64^

The DFT-optimized structure of [V^IV^O(pic)_2_(H_2_O)] was preliminary treated replacing the equatorial leaving H_2_O ligand with a dummy hydrogen atom according to what was recently established.^27,28,30^

To identify the possible RNase A binding sites for the V^IV^O species, relative solvent-excluded surface (SES) calculations^65^ were preliminarily performed focusing on the most exposed potential coordinating residues. The docking simulations were carried out constructing in the region of interest an evaluation sphere of 12 Å. Side-chain flexibility was taken into account considering the GOLD-implemented rotamer libraries.^66^ Genetic algorithm (GA) parameters have been set to 50 GA runs and a minimum of 100 000 operations. The other parameters of GA were set to default.

Scoring (Fitness of GoldScore) was evaluated applying the modified version of the GoldScore scoring function, which was validated in previously published papers.^27,28,30^ The best solutions (binding poses) were evaluated through three main criteria: (i) the mean (*F*_mean_) and the highest value (*F*_max_) of the scoring (Fitness of GoldScore) associated with each pose, (ii) the population of the cluster containing the best pose, and (iii) the position in the Fitness ranking of the computed cluster.

The refinement of the adduct found by dockings was performed by QM/MM ONIOM simulations defining the vanadium moiety and the coordinating side chain as the high layer. All the residues within a radius of 3.0 Å from the VC were treated as flexible. The geometry optimizations were performed using electronic embedding at the B3LYP/6-31g(d,p) level of theory including D3 Grimme’ correction for dispersion for the high level (BS1), while the AMBER99SB force field was applied for the low layer. The final energies, reported as relative qh-Gibbs (quasi-rigid-rotor-harmonic-oscillator),^67^ obtained with a frequency cutoff of 100 cm^–1^,^68^ were calculated adding to the thermal and entropic terms at BS1 the extrapolated energy using the basis set BS2, consisting of the triple-ζ def2-TZVP for main-group elements and the quadruple-ζ def2-QZVP for vanadium.

### Ribonucleolytic Activity Assays

The enzymatic activity of RNase A was measured by monitoring the cleavage of yeast RNA via UV–vis spectroscopy. The protein was incubated for 3 and 24 h in the presence of different concentrations of [V^IV^O(pic)_2_(H_2_O)] to reach final protein/metal ratios of 1/0.5, 1/1, 1/5, and 1/10. After incubation, its activity on yeast RNA was determined at room temperature in 0.050 M sodium acetate at pH 5.0, using 0.5 mg mL^–1^ RNA and an enzyme concentration of 7.3 μM. Under these experimental conditions, [V^IV^O(pic)_2_(H_2_O)] was stable in the time range explored in the assays. The enzymatic activity of native RNase A was also measured and used as a reference. Data are reported as the average of three independent measurements.

## Results and Discussion

### ESI-MS Spectrometry

To establish if the interaction of [V^IV^O(pic)_2_(H_2_O)] and RNase A occurs in aqueous solution, ESI-MS(+) spectra were recorded in an ammonium acetate solution (pH 6.4) and water (pH 5.4) varying the molar ratio between the VC and the protein (3/1–5/1) at an RNase A concentration of 5 μM. The pH does not change with the ratio, but it depends only on the used medium.

**Figure 1:**
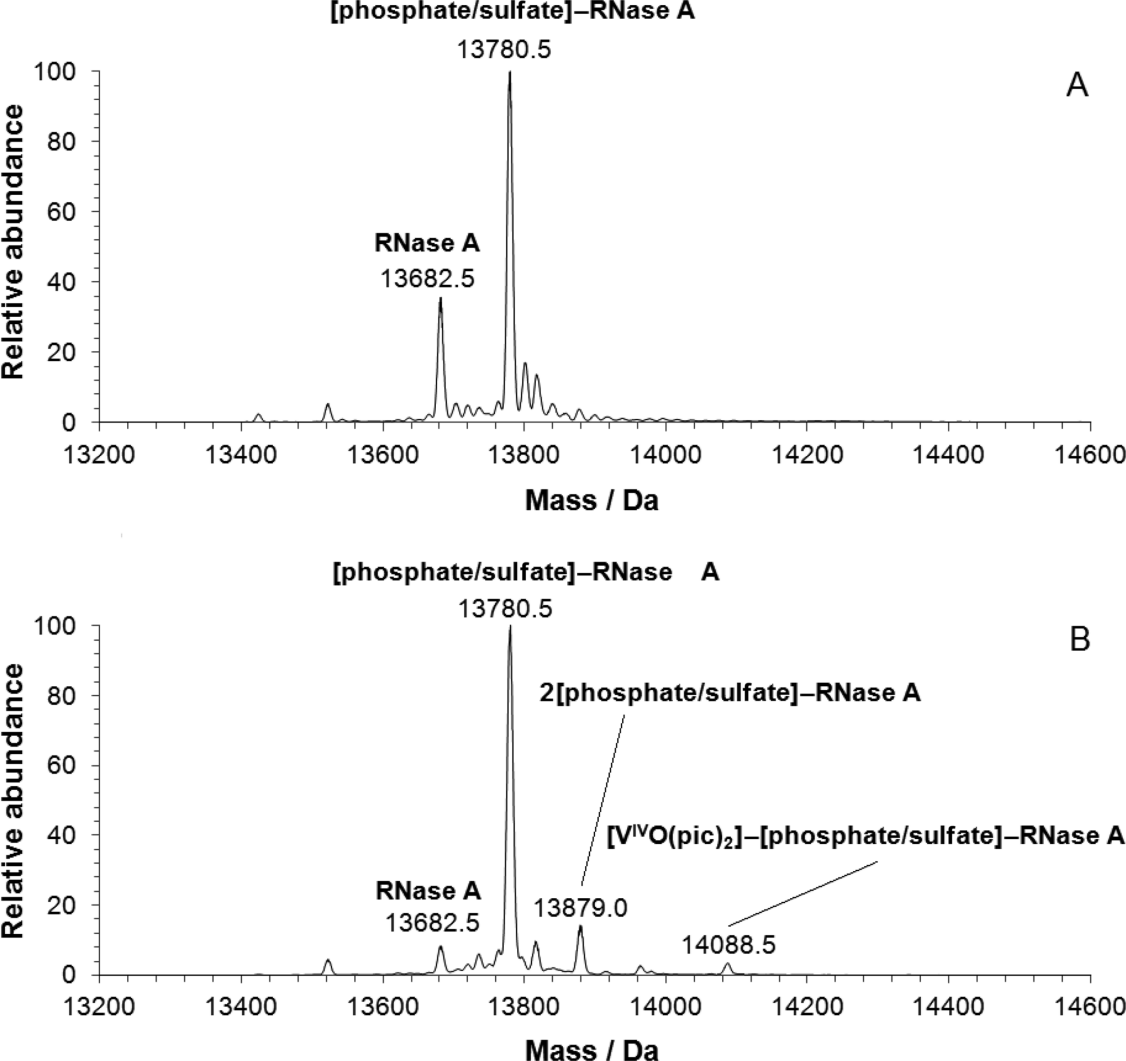
Deconvoluted ESI-MS(+) spectra of (A) RNase A and (B) RNase A in the presence of [V^IV^O(pic)_2_(H_2_O)] with a metal-to-protein molar ratio of 3/1. Both spectra were recorded in 1 mM ammonium acetate solution (pH 6.4) with a protein concentration of 5 μM.

The spectrum of free RNase A was recorded as a reference and is characterized by a series of peaks with different charged states of the protein from +7 to +10 (Figure S1A); the detected peaks for each state can be assigned to the adducts formed with phosphate/sulfate and Na^+^ or K^+^ ions. In the deconvoluted spectrum in NH_4_AcO buffer (Figure 1A), the signals of the free protein and of the adduct with phosphate or sulfate ions at 13 682.5 and 13 780.5 Da were detected and were attributed to RNase A and [phosphate/sulfate]–RNase A. This is not surprising considering the high affinity of RNase A for phosphate/sulfate ions^69^ and a possible small amount of phosphate or sulfate in the commercial protein. This finding and the spectral pattern are similar to those detected by Chowdhury et al. for metal-free RNase A and by Zoppi et al. in the system of RNase A with auranofin.^70^ When [V^IV^O(pic)_2_(H_2_O)] is added to the solution, the charge distribution of the protein peaks shifts to *z* values from +7 to +12, indicating an interaction with the metal species (Figure S1B). The deconvoluted spectrum shows the formation of the adduct [V^IV^O(pic)_2_]–[phosphate/sulfate]–RNase A, clearly observable at 14 088.5 Da, in a region where no peaks appeared in the spectrum of the free protein (Figure 1B). The spectra recorded in the system [V^IV^O(pic)_2_(H_2_O)]/RNase A in water at pH 5.4 are comparable (Figure S2). This result demonstrates that the interaction exists, even if no conclusions can be drawn about the strength of the binding. Data also suggest that the V compound binding does not affect the capability of the protein to bind the phosphate/sulfate ion. Notably, it is the moiety V^IV^O(pic)_2_ and not [V^IV^O(pic)_2_(H_2_O)] that interacts with the protein, in agreement with previous data in the literature.^12e,30^

### CD and UV–Vis Spectroscopy

Then, circular dichroism spectra of RNase A in the absence (time zero) and in the presence of the metal complex (protein-to-metal ratio of 1/3) in 10 mM sodium citrate buffer at pH 5.1 have been also collected in order to verify that the protein remains well-folded in the presence of the V compound (Figure S3). It has been demonstrated that the vanadium complex is stable under these experimental conditions, collecting UV–vis spectra of [V^IV^O(pic)_2_(H_2_O)] as a function of time in the absence and presence of the protein (Figure S4). CD spectra of RNase A in the presence of [V^IV^O(pic)_2_(H_2_O)] indicate that the enzyme retains its secondary structure in the presence of the metal complex, even when incubated with a large amount of the VC (up to a protein-to-metal ratio of 1/3).

Since literature data indicate that metalation can alter the stability of proteins,^71^ we also evaluated the thermal stability of RNase A in the presence of [V^IV^O(pic)_2_(H_2_O)]. Thermal shift assays carried out following the signal at 222 nm as a function of temperature for metal-free RNase A and for the protein in the presence of the VC suggest that also the overall stability of the protein is not influenced by the presence of the metal complex (Figure S3B).

### EPR Spectroscopy

EPR spectra were recorded in the system [V^IV^O(pic)_2_(H_2_O)]/RNase A both at 298 and 120 K. While the spectrum at room temperature of [V^IV^O(pic)_2_(H_2_O)] is isotropic,^27^ that collected with RNase A shows clear anisotropic components (Figure S5). This indicates an interaction between the metal complex and the protein because for large complexes such as the ternary adducts V^IV^O–ligand–protein, the rotational motion of the species in solution is slowed down.^27^

**Figure 2:**
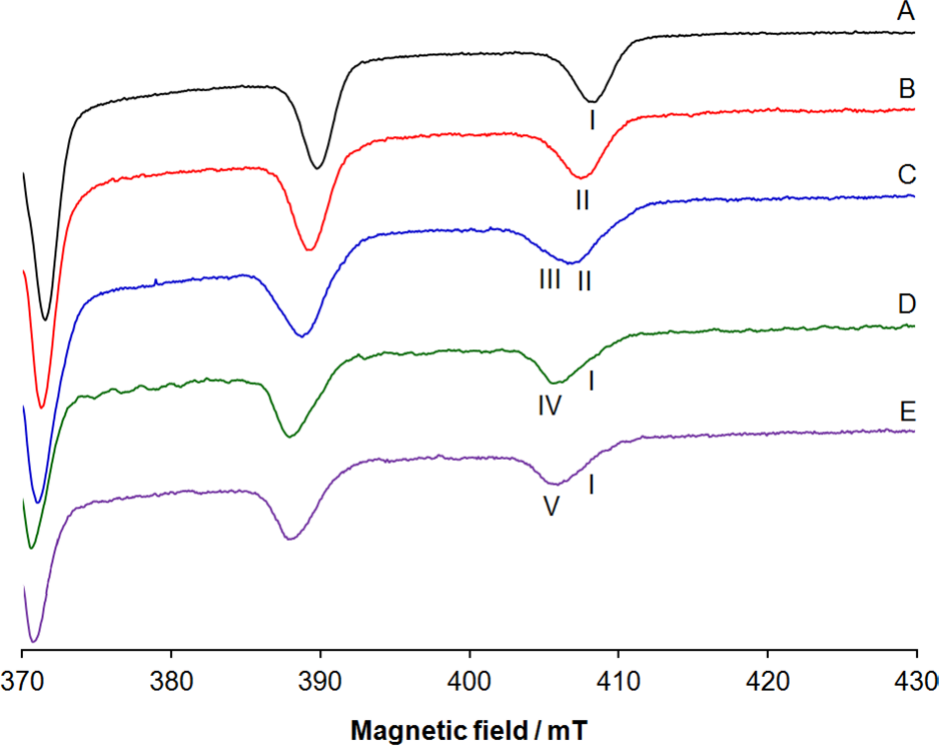
High-field region of anisotropic X-band EPR spectra recorded at 120 K in an aqueous solution containing the following: (A) [V^IV^O(pic)_2_(H_2_O)], V concentration of 1.0 mM; (B) [V^IV^O(pic)_2_(H_2_O)]/RNase A 1/3, pH 5.4, V concentration of 0.8 mM; (C) [V^IV^O(pic)_2_(H_2_O)]/RNase A 1/3, pH 7.4, V concentration of 0.8 mM; (D) [V^IV^O(pic)_2_(H_2_O)]/MeIm 1/4, pH 7.4, V concentration of 1.0 mM; (E) [V^IV^O(pic)_2_(H_2_O)]/IgG 1/1, pH 7.4, V concentration of 0.3 mM. **I** indicates the *M*_I_ = 7/2 resonances of [V^IV^O(pic)_2_(H_2_O)], **II** of the [V^IV^O(pic)_2_]–RNase A adduct with Asp/Glu-COO^–^ coordination, **III** of the [V^IV^O(pic)_2_]–RNase A adduct with His-N coordination, **IV** of the [V^IV^O(pic)_2_(MeIm)] complex, and **V** of the [V^IV^O(pic)_2_]–IgG adduct with His-N coordination. The number of scans was 5 for traces A–D and 10 for trace E.

The high-field region of the spectra at 120 K is shown in Figure 2. All the spectra were simulated with the software WINEPR SimFonia.^52^ The experimental and simulated signals of the full spectra are depicted in Figures S6–S10, the comparison between the experimental and simulated low-field and high-field regions is shown in Figures S11 and S12, and the spin Hamiltonian parameters are reported in Table S2; in addition, the instrumental settings are listed in the Supporting Information. It should be noticed that the spectra were simulated with *g_x_* = *g_y_* and *A_x_* = *A_y_*, i.e., assuming a tetragonal symmetry (Table S2); this is perfectly in line with the results in the literature, which showed that the parameter related to *x*,*y* anisotropy, |*A_x_* – *A_y_*|, is very small for an octahedral V^IV^O species, usually less than 2–3 × 10^–4^ cm^–1^.^47,72^

When the signals of [V^IV^O(pic)_2_(H_2_O)] with coordination (N, COO^–^), (N, COO^–*ax*^), and H_2_O (**I** in Figure 2A) are compared with those collected with the protein at different pHs, a significant variation of the hyperfine coupling constant along the *z* axis, *A_z_*(^51^V), is revealed. The value of *A_z_*(^51^V) goes from 164.6 × 10^–4^ cm^–1^ for [V^IV^O(pic)_2_(H_2_O)]^47^ to 163.0 × 10^–4^ cm^–1^ in the system with RNase A at pH 5.4 (Figure 2B), i.e., close to the conditions used to carry out the X-ray diffraction (XRD) analysis (*videinfra*), and to 159.0 × 10^–4^ cm^–1^ in the system with RNase A at pH 7.4 (Figure 2C). This variation denotes a change in the equatorial donors bound to V^IV^ and, in particular, the replacement of the equatorial water-O in [V^IV^O(pic)_2_(H_2_O)] with Asp-Glu/COO^–^ at acidic pH and His-N at physiological pH, which, according to “additivity relationship” that allows estimation of *A*_z_(^51^V) from the contribution of the four donors in the equatorial plane of the V^IV^O^2+^ ion,^73^ should result in an observable reduction of the hyperfine coupling constant. In fact, such a rule predicts that an Asp/Glu-COO^–^ donor should cause a decrease in *A_z_*(^51^V) of about 2–3 × 10^–4^ cm^–1^ and a His-N of 5–6 × 10^–4^ cm^–1^ in comparison with water-O.^73^ Notably, the value of *A_z_*(^51^V) for the adduct [V^IV^O(pic)_2_]–RNase A with Asp/Glu-COO^–^ coordination (resonances indicated with **II** in Figure 2) is in line with [V^IV^O(pic)_2_]–lysozyme (163.0 × 10^–4^ cm^–1^^30^), while that of [V^IV^O(pic)_2_]–RNase A with His-N coordination (**III**) is comparable with [V^IV^O(pic)_2_(MeIm)]^16^ (159.0 × 10^–4^ cm^–1^, **IV**; Table S2) or [V^IV^O(pic)_2_]–IgG^51f^ (159.8 × 10^–4^ cm^–1^, **V**; Table S2).

From a chemical point of view, the variation of the EPR signals can be explained assuming that at pH 5.4, the His nitrogens are protonated and only the carboxylate groups are able to bind V^IV^, while at pH 7.4, His-N, in the deprotonated form, can partly replace the Asp/Glu-COO^–^ group due to its higher basicity.

### X-ray Structure

**Figure 3:**
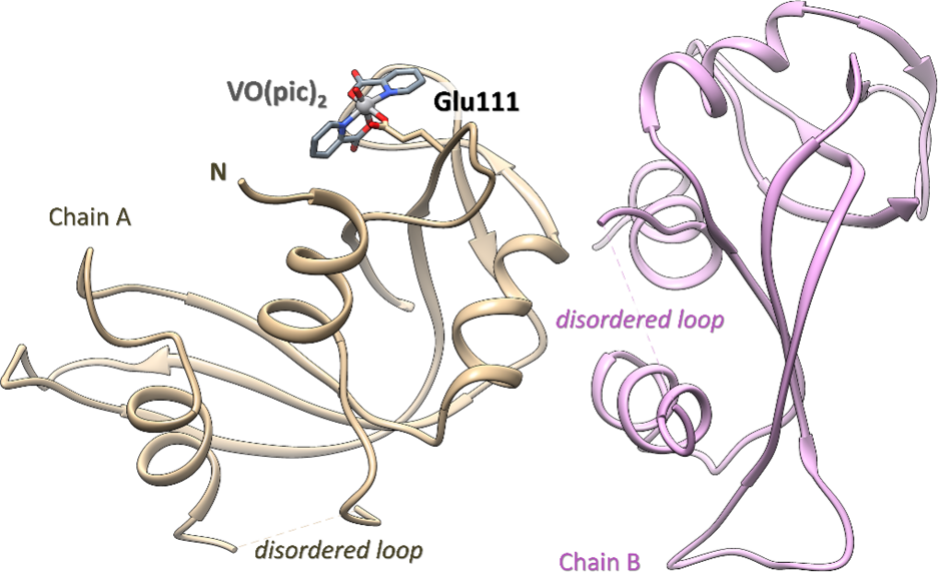
Structure of the [V^IV^O(pic)_2_]–RNase A adduct (PDB code: 7P8R). The two molecules in the asymmetric unit are reported in tan and purple. The V binding site is close to the side chain of Glu111 of molecule A.

Crystals of the adduct formed upon the reaction of [V^IV^O(pic)_2_(H_2_O)] with RNase A were then obtained using the soaking strategy: crystals of the metal-free protein grown in 22% PEG4K and 10 mM sodium citrate at pH 5.1 using a protein concentration 1.4 mM have been soaked for 3 days in a solution of the reservoir saturated with the metal complex. The X-ray structure of the adduct was refined using diffraction data up to 1.27 Å (*R*-factor, 0.158 and R-free, 0.222). The structure presents two molecules in the asymmetric unit, denoted as molecule A and molecule B hereafter, and consists of 2420 non-hydrogen atoms, including two phosphate/sulfate ions that are located in the active site of two protein molecules of the asymmetric unit. The electron density map of the protein molecules is well-defined with exception of the region that encompasses residues 20 and 21 of molecule A and residues 17–21 of molecule B, which have been not included in the final model (Figure 3).

**Figure 4:**
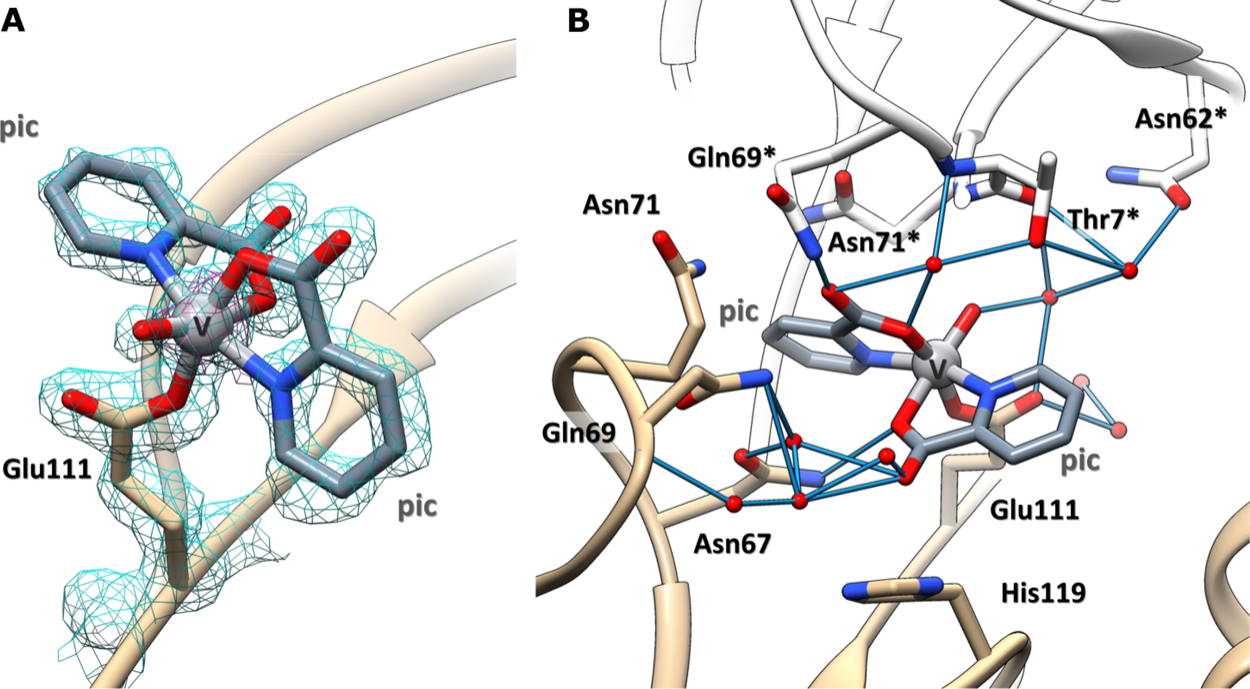
(A) Details of the VC binding site in the structure of the [V^IV^O(pic)_2_]–RNase A adduct formed upon the reaction of [V^IV^O(pic)_2_(H_2_O)] with the protein. The V atom binds the OE1 atom of the side chain of Glu111, which replaces the water molecule of [V^IV^O(pic)_2_(H_2_O)]. 2Fo–Fc electron density maps are contoured at the 1.0σ level (cyan) and the 5.0σ level (black). The anomalous differences in the electron density map, which allow the unambiguous determination of the V position, are reported in purple at the 3.0σ level. (B) Interaction of the V complex fragment with the surrounding water molecules and protein residues of molecule A and from a symmetry-related molecule (molecule B, indicated with the asterisks). The alternative conformation of the side chain of His119 was omitted for the sake of clarity.

Data collection and refinement statistics for this structure are reported in Table S1. Inspection of the Fo–Fc electron density maps reveals that the V complex binds to molecule A, while molecule B remains unaltered. This happens because the VC binding site of molecule B is hampered by crystal packing. Thus, the comparison of the structures of molecules A and B furnishes a detailed picture of the structural modifications associated with the V complex binding. The superimposition of the two protein molecules in the asymmetric unit of the adduct indicates that the binding of the VC induces only small variations in the structure of the protein. In fact, the two molecules present a root-mean-square deviation (r.m.s.d.) of the Cα atom of 0.43 Å. The most significant differences observed in the two protein molecules are the conformations of the side chains of surface residues. The V complex binds the side chain of Glu111 (Figure 4), with occupancy = 0.75 and the *B*-factor within the range of 13.1–23.2 Å^2^. The presence of the V center has been confirmed by inspection of the anomalous difference electron density map (Figure S13). Interestingly, although RNase A has been used as a model system in several metalation studies,^38−46^ Glu111 was never identified as a metal binding site. This result depends both on the accessibility of the Glu111 residue and on its affinity for the “hard” V^IV^ ion. At the metal complex binding site, V adopts a slightly distorted octahedral geometry, with a bidentate coordination of the two picolinato ligands, an O oxido atom and the OE1 (or oxygen Oγ) from Glu111 completing the metal coordination sphere (Figure 4A). The V^IV^=O bond length in our structure is 1.68 Å, which is slightly larger than the expected value (1.57–1.65 Å),^74^ while the V^IV^–O(pic) bond lengths are 1.80 and 2.03 Å, in agreement with expectations.^74^ Notably, a long V^IV^=O bond was observed also in the X-ray structure of the adduct of [V^IV^O(pic)_2_(H_2_O)] with the HEWL;^24^ this observation was explained considering a possible reduction of V^IV^ to V^III^ due to crystal exposure to the X-ray beam.^24^ The distance between V^IV^ and the OE1 atom of Glu111 is 1.97 Å. The binding of the V complex is stabilized by an intricate network of H-bonds formed with water molecules. At the solid state, additional stabilizations come from contacts with residues from a symmetry-related molecule (Figure 4B). It is important to note that, among the two stable isomers of the V^IV^O(pic)_2_ moiety and their enantiomers, *OC*-6-23-Δ/Λ and *OC*-6-24-Δ/Λ,^30,47,48^ only *OC*-6-23-Δ (O donor *trans* to water, Scheme S1) was found to coordinate the protein, highlighting a high degree of chiral discrimination of the binding region.

### Computational Studies

To gain insight into the recognition factors governing both the specificity of the binding region and its chiral discrimination, a multiscale computational strategy was applied. First, the binding region selectivity was rationalized by combining docking and QM/MM simulations. Then, state-of-the-art DFT-based noncovalent interaction (NCI) analysis unveiled the secondary interaction network behind the chiral discrimination of the binding site.

**Figure 5:**
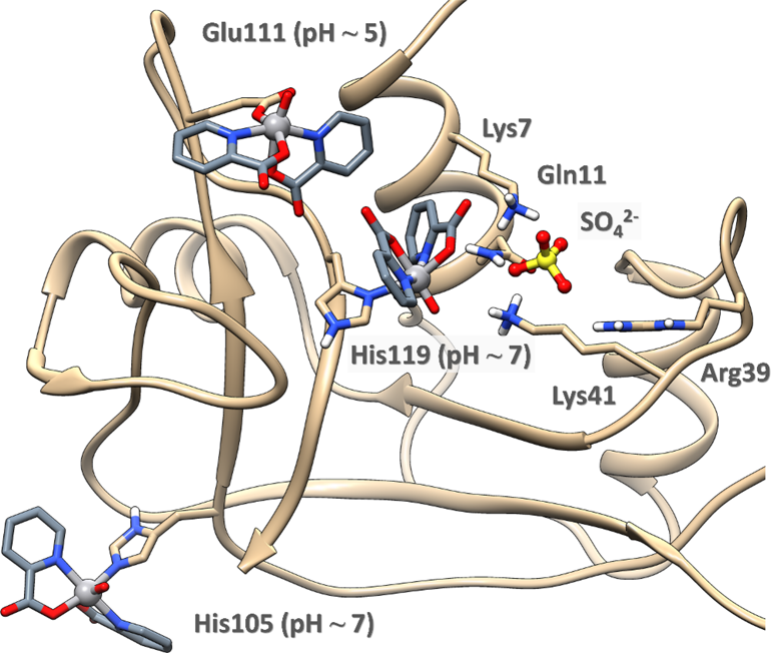
Binding of the moiety *OC*-6-23-Δ-V^IV^O(pic)_2_ to the residues Glu111, His105, and His119, as determined by docking and QM/MM calculations.

Taking into account the EPR data at different pHs (Figure 2), the binding of carboxylate- or imidazole-containing side chains appears to be possible at pHs 5.4 and 7.4, respectively. The carboxylate donor was clearly identified by XRD data (Glu111, Figure 4), while for the imidazole-containing residues, the protein surface was probed for the well-exposed histidines able to coordinate the V^IV^O(pic)_2_ moiety, allowing us to identify His105 and His119 as putative coordinating residues at physiological pH (Figure 5). Docking analysis on these regions for the two favored isomers and the respective enantiomers of the V^IV^O(pic)_2_ moiety, which are *OC*-6-23-Δ/Λ and *OC*-6-24-Δ/Λ with the two nitrogen atoms in the equatorial plane of the V^IV^O^2+^ ion (see Scheme S1 and refs (30), (47), and (48)), confirmed the possibility to coordinate Glu111 as well as His105 and His119, with the highest affinity shown by the *OC*-6-23-Δ isomer for all the regions, in perfect agreement with XRD data. The highest-affinity docking structures were refined by QM/MM calculations obtaining Δ*G*_gas_ values in the order Glu111 (−57.7 kcal·mol^–1^, pH ∼ 5) ≫ His119 (−18.8 kcal·mol^–1^, pH ∼ 7) > His105 (−13.8 kcal·mol^–1^, pH ∼ 7), see Table S3. It is important to note that His119 is involved in the P1 subsite of the enzyme that stabilizes, with His12 and Lys41, the interaction with sulfate/phosphate ions.^69b^ These results suggest that (i) His105 should be preferred compared to His119 that displays a lower accessibility since it is involved in the sulfate (or phosphate) stabilization in the P1 subsite of the enzyme, as observed by XRD and ESI-MS data; (ii) in principle, the enzyme inhibition could also take place at physiological pH upon binding of His119 to V^IV^ (*vide infra*). The most probable binding sites are shown in Figure 5. This finding is consistent with the EPR results, which indicate that at acidic pH, when the His residues are protonated, the unique coordination is through carboxylate-containing donors, while at neutral pH, with the His residues available for coordination, resonances compatible with an equatorial imidazole binding were observed. The preferential binding of V^IV^O(pic)_2_ to only one (His105) or two His residues (His105 and His119) accounts for the lower relative intensity of the EPR signal of the adduct formed by RNase A at physiological pH (see Figure 2) with respect to that, for example, of [V^IV^O(pic)_2_]–IgG^51f^ (**V** in Figure 2) and [V^IV^O(pic)_2_]–Hb^75^ in which the binding of histidines is favored; for comparison, IgG has 12 surface His residues,^76^ and Hb has 26 His residues.^77^

**Figure 6:**
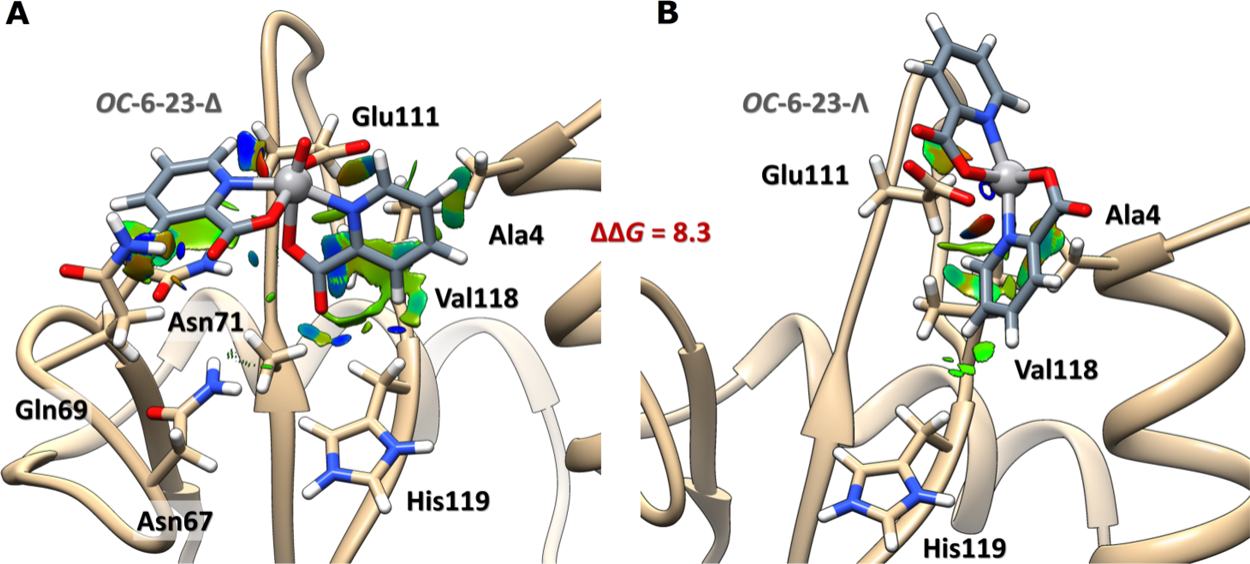
Intermolecular gradient isosurface (*s* = 0.3 a.u.) analysis of the better solution for binding of (A) *OC*-6-23-Δ-V^IV^O(pic)_2_ and (B) *OC*-6-23-Λ-V^IV^O(pic)_2_ to Glu111 at pH ∼ 5. The surfaces are reported in a blue–green–red scale according to values of sign(λ_2_) × ρ. Blue surfaces indicate strong attractive interactions (such as dipole–dipole or H-bond), red indicates repulsion, and green means vdW interactions. The ΔΔ*G* value in the gas phase is reported in kcal·mol^–1^.

The factors governing the chiral selectivity of the binding were identified by NCIPlot (noncovalent interactions plot^78,79^) in an analysis which unveils that only the *OC*-6-23-Δ structure can be coordinated by the COO^–^ group of Glu111 establishing an extended H-bond network and vdW stabilizations. In contrast, the Λ isomer needs to rotate ∼90° with respect to the V^IV^–O axis to avoid steric clashes, thus breaking the favorable interactions observed for the Δ series (Figure 6).

As discussed for the molecular structure of the adduct, the specificity and selectivity of the binding site stand on (i) direct H-bonds through Asn69 and Asn71 with the carboxylate group of the picolinato ligands; (ii) the additional H_2_O-mediated H-bond between Asn67 and the carboxylate group of pic^–^; (iii) the stabilization given by π stacking and CH−π vdW interactions between the amide functionality of Asn71 and one pic^–^, plus the second pic^–^ ligand with Val118 and Ala4.

### Ribonucleolytic Activity Assays

We have also investigated the ability of [V^IV^O(pic)_2_(H_2_O)] to bind RNase A in solution by studying the catalytic activity of the protein in the presence of increasing amounts of the V complex and as a function of incubation time of the protein with the potential metallodrug. RNase A catalyzes the breakdown of RNA by means of transesterification from the 5′ position of one nucleotide to the 2′ position of the adjacent nucleotide with the formation of a 2′,3′-cyclic phosphate product that is irreversibly hydrolyzed to a 3′ nucleotide.^69b^ Results of the catalytic activity assays, carried out in 50 mM sodium acetate at pH 5.0, demonstrate that the protein is significantly inhibited in the presence of the VC, even with a protein-to-metal molar ratio of 1/1 and after just 3 h of incubation (Figure S14). The inhibition is not dependent on the metal complex concentration, as already observed in many other studies on metalation of the protein.^41,42,45,46^ It is probable that this behavior may be due to the finding that, under the investigated experimental conditions, the protein is not fully metalated, and thus, the same portion of the protein is inhibited at different concentrations of the V complex.

**Figure 7:**
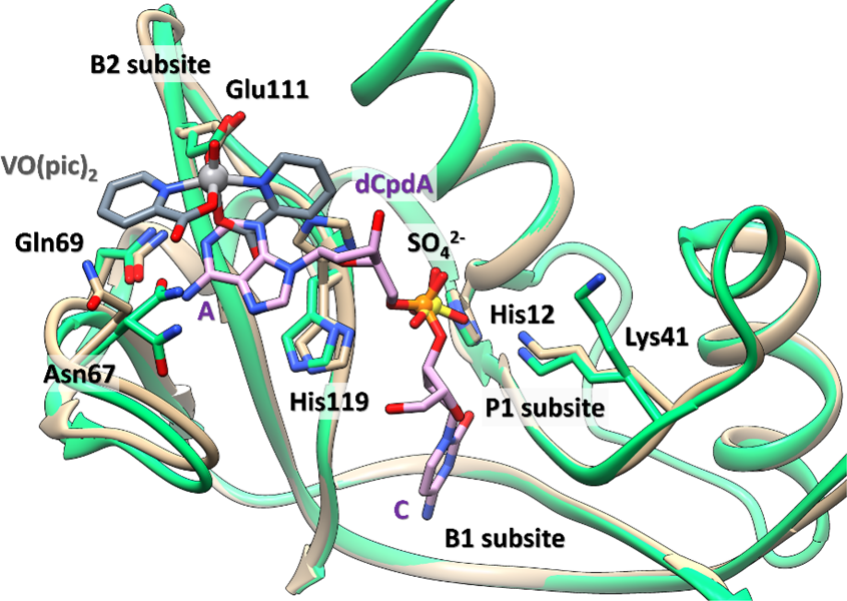
Details of the structure of the adduct formed upon the reaction of [V^IV^O(pic)_2_(H_2_O)], in gray, with RNase A (tan) superposed on the structure of the complex of RNase A with d(CpA), in purple, which is a substrate analogue (PDB code: 1RPG; green^81^). The VC obstructs the B2 subsite (Asn67, Asn71, Gln69, and Glu111), where the adenine base (“A” in the figure) is accommodated. “C” stands for cytosine that occupies the B1 subsite (Thr45, Phe120, and Ser123). The residues constituting the P1 subsite (His12, His119, and Lys41) are also indicated.

The results of the catalytic activity assays are well-explained by the X-ray structure. The RNase A active site is constituted by a P1 subsite,^69^ which hosts the hydrolyzable phosphodiester group and is composed by His12, His119, and Lys41 (catalytic triad), and two subsites, B1 and B2, that preferentially bind a pyrimidine and a purine base, respectively.^80^ In the structure of the adduct formed upon the reaction of RNase A with [V^IV^O(pic)_2_(H_2_O)], the VC is close to the protein active site, at 4.1 Å from the catalytically competent conformation of the side chain of His119 (Figure 7). Notably, the B2 subsite of the protein is obstructed by the presence of the V complex, as evidenced by superimposing our structure with that of RNase A complexed with the deoxycytidyl-3′,5′-deoxyadenosine dinucleotide [d(CpA)] (PDB code: 1RPG(81)). This well accounts for the reduced catalytic activity of the protein in the presence of [V^IV^O(pic)_2_(H_2_O)].

## Conclusions

Despite the central role of proteins in the transport of V-based drugs, their interaction with V complexes (in particular V^IV^) has been still scarcely investigated, in particular from the structural point of view.^13^ Moreover, the X-ray structures of (V drug)–protein adduct are still scarce compared to other biologically active metals. On the other hand, recent developments in theoretical techniques were achieved to predict the covalent and noncovalent binding of a vanadium moiety to a protein; such an approach, combined with spectrometric and spectroscopic methods, has been also applied to the VCs–protein systems with encouraging results.^29^

Here, we have studied the interaction of [V^IV^O(pic)_2_(H_2_O)] with RNase A by a combination of biophysical techniques, including X-ray crystallography. While spectrometric and spectroscopic techniques, such as ESI-MS, CD, UV–vis, and EPR, allow us to unambiguously demonstrate the V–protein interaction, the XRD analysis allows us to obtain a detailed description of the three-dimensional structure of the adduct. The structure reveals that (a) [V^IV^O(pic)_2_(H_2_O)] binds RNase A with the metal center anchored to the side chain of Glu111, which replaces the water ligand; (b) the moiety V^IV^O(pic)_2_ binds to RNase A without altering the overall protein conformation and stability; (c) the structural features of binding regions induce the enantiomeric selectivity toward the *OC*-6-23-Δ isomer, with the selectivity depending on the favorable H-bond network and vdW stabilizations; (d) [V^IV^O(pic)_2_(H_2_O)] binding affects the catalytic activity of the enzyme by obstructing the B2 subsite.

Computational methods confirm the experimental findings, in particular the coordination of Glu111 at acidic pH and His105 and/or His119 at neutral pH. These results demonstrate once again the prediction capability of the theoretical approach, which could be used for a valuable prediction of VCs–protein interactions in the absence of a structural determination.

The binding of V significantly affects the enzymatic activity of RNase A, even it does not alter its stability. So, we have demonstrated that also V^IV^ complexes, in addition to mono- and polynuclear vanadate(V), are able to inhibit RNase A.^82,83^

Overall, the data indicate that the binding of VCs–protein is possible in various modes and under various conditions and that the interaction may be related to the mechanism of action and formation of the active species. The possibility of an inhibition of cytosolic proteins upon the V binding must be also considered. These results could be important for the design and development of V complexes as therapeutic agents.

## Supporting Information Available

Tables with X-ray diffraction data collection and refinement statistics (Table S1), spin Hamiltonian EPR parameters (Table S2), and computational results (Table S3); figures with ESI-MS, UV–vis, CD, and EPR spectra (Figures S1–S12), electron density map (Figure S13), and catalytic activity assays (Figure S14); scheme with the isomers of [V^IV^O(pic)_2_(H_2_O)] (Scheme S1) (PDF)
