# Supplementary material for: Spectroscopic/Computational Characterization and the X-ray Structure of the Adduct of the VIVO–Picolinato Complex with RNase A
Source: Inorg Chem. 2021 Nov 30;60(24):19098–109. doi: 10.1021/acs.inorgchem.1c02912 (PMC8693189; doi:10.1021/acs.inorgchem.1c02912)
Supplement: Supplementary file 1 — ic1c02912_si_001.pdf [file ic1c02912_si_001.pdf]

SUPPORTING INFORMATION

Spectroscopic/computational characterization and the X-ray structure of the adduct of the $V^{IV}O$–picolinato complex with RNase A

Giarita Ferraro,[a] Nicola Demitri,[b] Luigi Vitale,[a] Giuseppe Sciortino,[c] Daniele Sanna,[d] Valeria Ugone,[d] Eugenio Garribba,*[e] Antonello Merlino,*[a]

[a] Department of Chemical Sciences, University of Naples Federico II, I-80126 Napoli, Italy

[b] Elettra–Sincrotrone Trieste, S.S. 14 Km 163.5 in Area Science Park, Trieste, Italy

[c] Institute of Chemical Research of Catalonia (ICIQ), The Barcelona Institute of Science and Technology, 43007 Tarragona, Spain

[d] Istituto di Chimica Biomolecolare, Consiglio Nazionale delle Ricerche, Trav. La Crucca 3, I-07100 Sassari, Italy

[e] Dipartimento di Scienze Mediche, Chirurgiche e Sperimentali, Università di Sassari, I-07100 Sassari, Italy

Corresponding authors. E-mail: garribba@uniss.it (E.G.); antonello.merlino@unina.it (A.M.).

Table S1. Data collection and refinement statistics of the structure of the adduct formed upon reaction of $[V^{IV}O(pic)_2(H_2O)]$ with RNase A.

Data collection	
Space group	C2
a (Å)	100.23
b (Å)	33.02
c (Å)	72.71
α/β/γ (°)	90.0/90.2/90.0
Resolution range (Å)[a]	72.71 - 1.27 (1.29-1.27)
Observations	289576 (10426)
Unique reflections	58090 (2507)
Completeness (%)	91.3 (79.8)
Redundancy	5.0 (4.2)
R_{merge} (%) [b]	0.095 (0.664)
Average I/σ(I)	8.6 (2.1)
$CC_{1/2}$	0.992 (0.828)
Anom. completeness (%)	89.1 (75.4)
Anom. Multiplicity	2.6 (2.2)
Wilson B-factor ($Å^2$)	12.1
Refinement	
Resolution range (Å)	72.71-1.27
N. of reflections (working set)	55036
N. of reflections (test set)	2754
R-factor/R-free (%)	15.8/22.2
N. of residues	250
N. of atoms	2420
Average B-factors ($Å^2$)	
All atoms	20.67
V atom	17.46
V complex occupancy	0.75
R.m.s. deviations	
Bond lengths (Å)	0.013
Bond angles (°)	1.67
Ramachandran statistics (Validation Report)	
Favoured	96.0%
Allowed	4.0%
Disallowed	0%

[a] Criteria used in determination of resolution cut: R_{pim} <= 0.6000; I/σ(I) >= 2.00; $CC_{1/2}$ >= 0.3000.

[b] $R_{merge} = \Sigma_h \Sigma_i |I_{(h,i)} - \langle I_{(h)} \rangle| / \Sigma_h \Sigma_i I_{(h,i)}$, where $I_{(h,i)}$ is the intensity of the ith measurement of reflection h and $\langle I_{(h)} \rangle$ is the mean value of the intensity of reflection h.

Table S2. Spin Hamiltonian EPR parameters of the $V^{IV}O$ species.

Complex	g_x	g_y	g_z	A_x [a]	A_y [a]	A_z [a]
$[V^{IV}O(pic)_2(H_2O)]$ [b]	1.981	1.981	1.947	-59.0	-59.0	-164.6
$[V^{IV}O(pic)_2]$–RNase A (Asp/Glu binding) [c]	1.982	1.982	1.949	-58.9	-58.9	-163.0
$[V^{IV}O(pic)_2]$–RNase A (His binding) [d]	1.979	1.979	1.951	-54.0	-54.0	-159.6
$[V^{IV}O(pic)_2(MeIm)]$ [e]	1.979	1.979	1.950	-53.8	-53.8	-159.0
$[V^{IV}O(pic)_2]$–IgG [f]	1.979	1.979	1.950	-54.1	-54.1	-159.8

[a] Values reported in 10^{-4} cm^{-1}. [b] Species indicated with **I** in Fig. 2 of the main text. [c] Species indicated with **II** in Fig. 2 of the main text. [d] Species indicated with **III** in Fig. 2 of the main text. [e] Species indicated with **IV** in Fig. 2 of the main text. [f] Species indicated with **V** in Fig. 2 of the main text.

Table S3. Docking and QM/MM binding solutions for the binding of the Λ and Δ isomer series of $V^{IV}O(pic)_2$ with RNase A.

	Docking							QM/MM	
Isomer	Donor	V···D[a]	F_{max}[b]	F_{mean}[c]	Interactions	Pop.[d]	Rank.[e]	V···D[a]	ΔG_{gas}[f]
***OC*-23-Δ (pH 5)**	Glu111	1.927	38.8	31.8	Gln81-NH_2···CO(pic)	50	I	1.966	-57.7
OC-23-Λ (pH 5)		2.319	27.2	26.4	–	48	I	2.363	-49.4
OC-24-Δ (pH 5)		2.227	28.2	27.2	–	47	I	–	–
OC-24-Λ (pH 5)		2.407	30.6	29.4	–	48	I	–	–
***OC*-23-Δ (pH 7)**	His119	2.365	45.9	41.7	π-stacking with pic	50	I	2.313	-18.2
OC-23-Λ (pH 7)		2.231	42.1	39.6	Lys7-NH_3···CO(pic)	48	I	–	–
OC-24-Δ (pH 7)		2.257	43.1	38.9	π-stacking with pic	41	I	–	–
OC-24-Λ (pH 7)		2.360	45.0	41.6	π-stacking with pic	22	I	–	–
***OC*-23-Δ (pH 7)**	His105	2.191	38.9	37.8	Gln74-NH_2···V=O	50	I	2.149	-13.2
OC-23-Λ (pH 7)		2.068	35.9	35.0	–	50	I	–	–
OC-24-Δ (pH 7)		2.252	35.7	34.4	–	50	I	–	–
OC-24-Λ (pH 7)		2.256	34.8	33.4	–	47	I	–	–

[a] Distance between V and the protein donor (D) in Å. [b] GoldScore *Fitness* value obtained for the most stable pose of each cluster (F_{max}). [c] Average value of GoldScore *Fitness* for each cluster (F_{mean}). [d] Population of the cluster (numbers of solutions per cluster). [e] Ranking of the identified cluster. [f] QM/MM ΔG_{gas} values at the theory level B3LYP-D3/BS2.

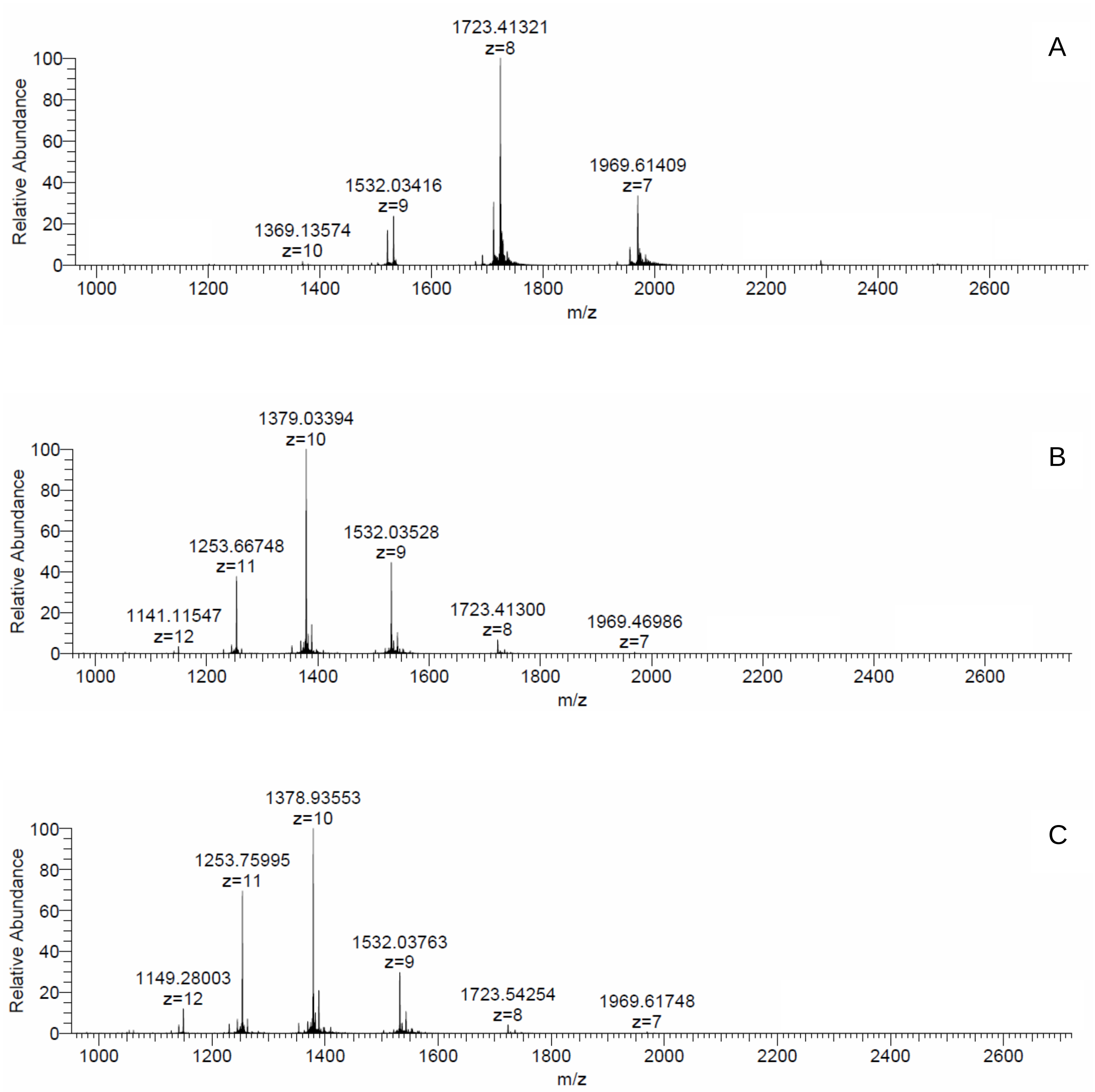

Fig. S1. ESI MS(+) spectra of: A) RNase A in a 1 mM ammonium acetate solution (pH 6.4); B) RNase A in the presence of [$V^{IV}O(pic)_2(H_2O)$] with a metal to protein molar ratio of 3/1 in a 1 mM ammonium acetate solution (pH 6.4); C) RNase A in the presence of [$V^{IV}O(pic)_2(H_2O)$] with a metal to protein molar ratio of 3/1 in water (pH 5.4). In all the spectra the protein concentration is 5 μM.

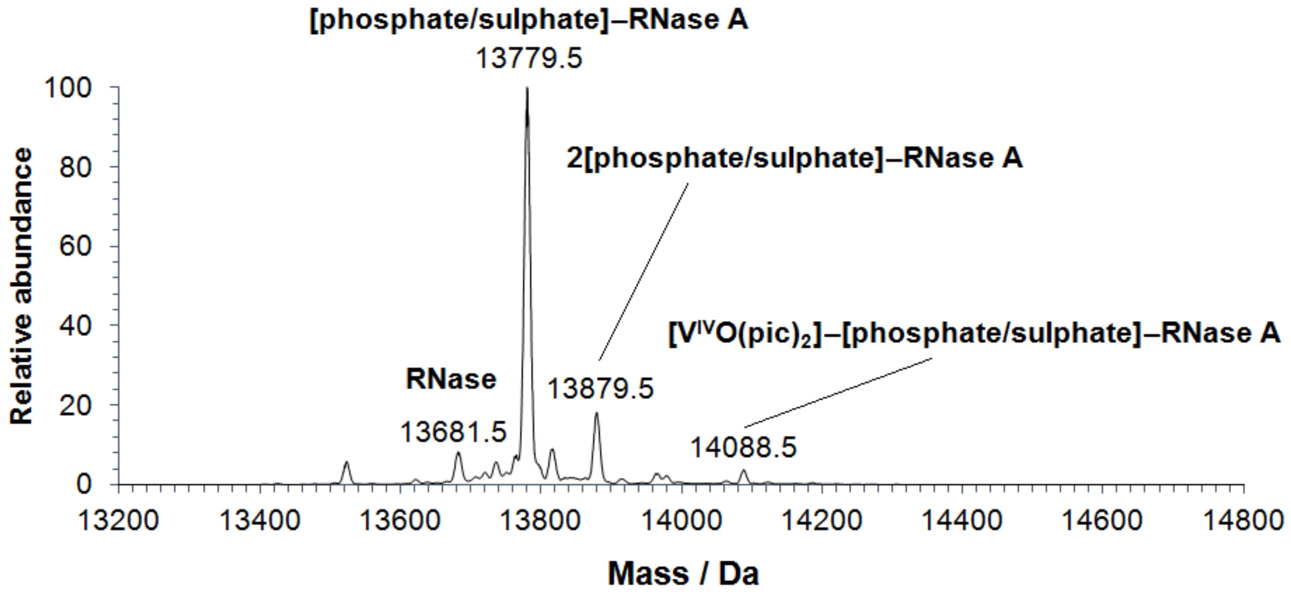

Fig. S2. Deconvoluted ESI-MS(+) spectrum of RNase A in water (pH 5.4) in the presence of [$V^{IV}O(pic)_2(H_2O)$] with a metal to protein molar ratio of 3/1 and a protein concentration of 5 µM.

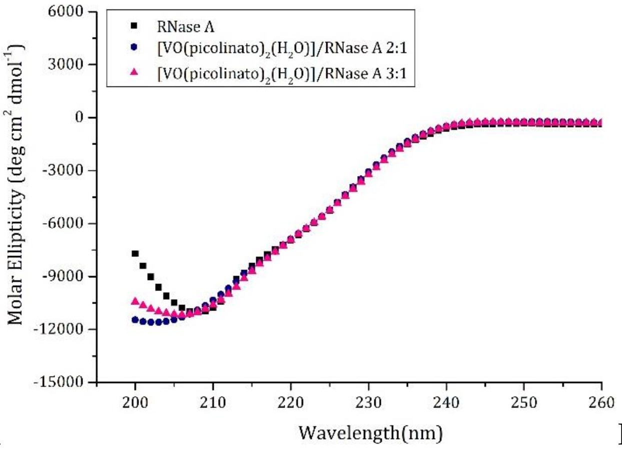

A

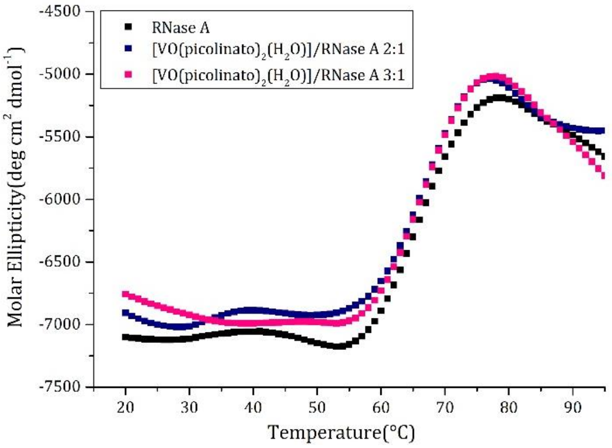

B

Fig. S3. (A) Circular dichroism spectra of the adduct formed upon reaction of RNase A with [$V^{IV}O(pic)_2(H_2O)$] at different protein to metal ratio compared to the spectrum of the metal-free protein (black squares) in 10 mM sodium citrate buffer pH 5.1 after 24 h of incubation at 20 °C. (B) Thermal denaturation profiles of the adduct formed upon reaction of [$V^{IV}O(pic)_2(H_2O)$] with RNase A, compared to that of native protein.

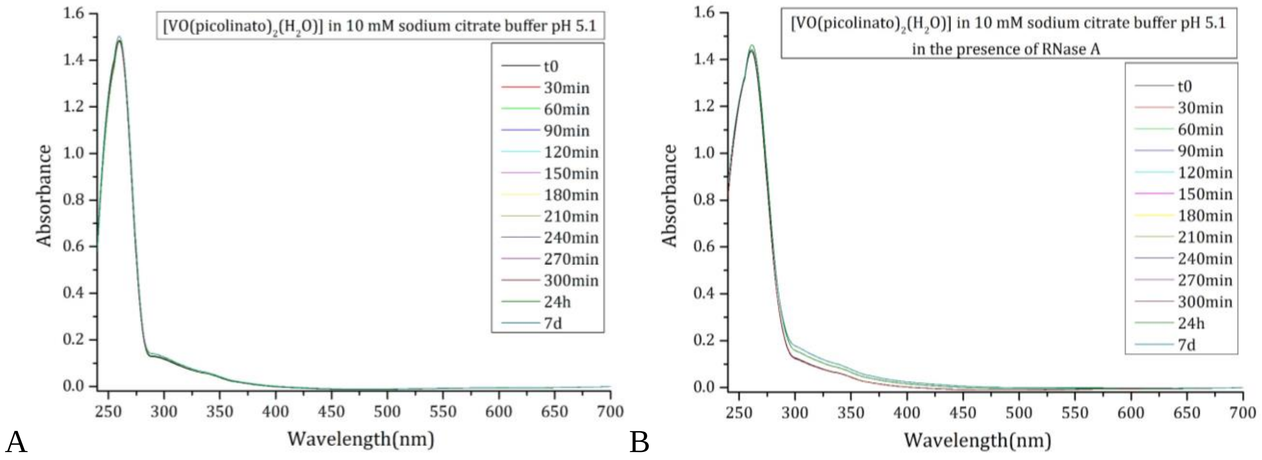

Fig. S4. UV-vis spectra of [$V^{IV}O(pic)_2(H_2O)$] (150 μM) in 10 mM sodium citrate buffer ay pH 5.1 as function of time in the absence (A) and in the presence (B) of RNase A (50 μM).

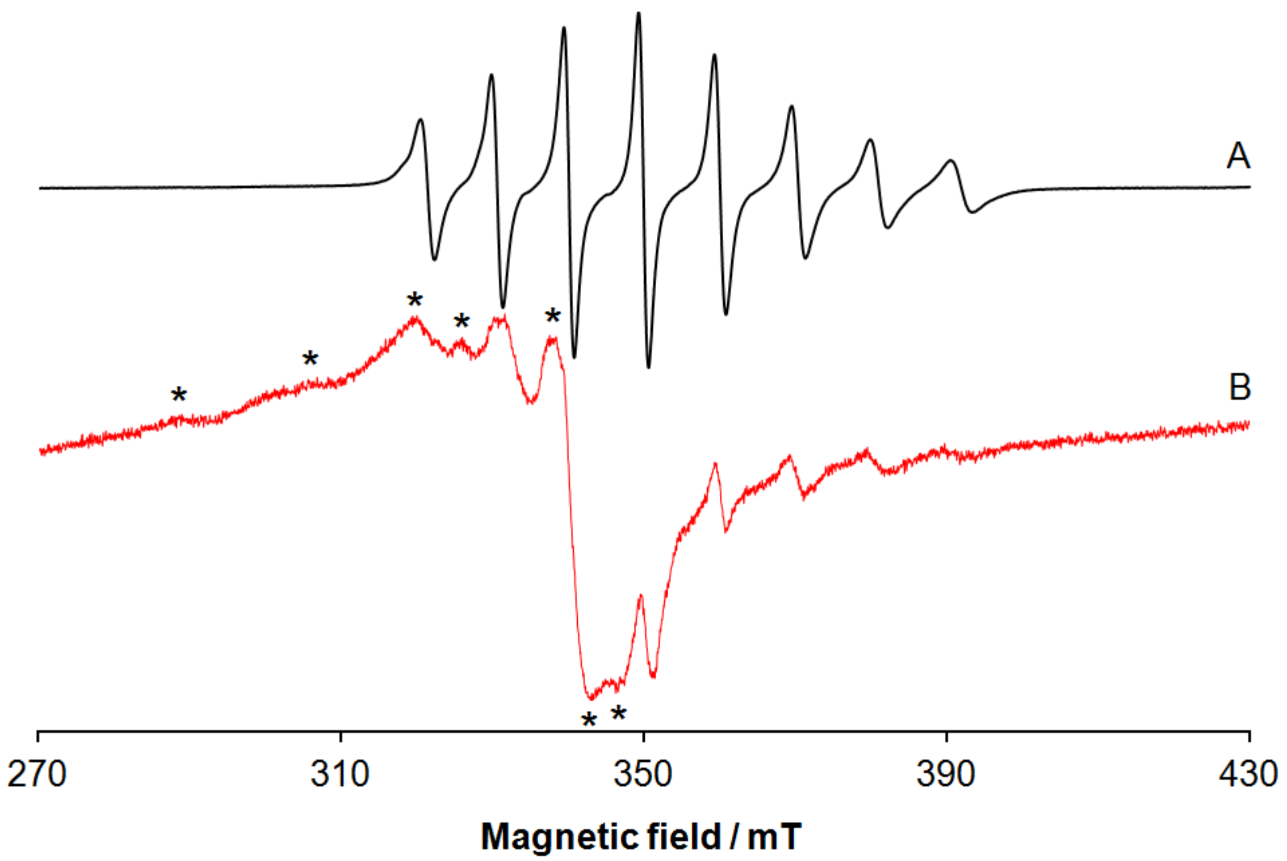

Fig. S5. X-band EPR spectra recorded at 298 K on aqueous solution containing: A) [$V^{IV}O(pic)_2(H_2O)$] (microwave frequency 9.853 GHz, microwave power 20.1 mW, modulation frequency 100 kHz, modulation amplitude 0.4 mT, time constant 81.92 ms, sweep time 335.5 s, resolution 4096 points); B) [$V^{IV}O(pic)_2(H_2O)$] and RNase A in metal to protein molar ratio 1/3, pH 7.4 (microwave frequency 9.404 GHz, microwave power 20.3 mW, modulation frequency 100 kHz, modulation amplitude 0.4 mT, time constant 40.960 ms, sweep time 335.5 s, resolution 2048 points). $V^{IV}O^{2+}$ concentration is 0.8 mM. With the asterisks the anisotropic resonances attributed to the [$V^{IV}O(pic)_2$]–RNase A adduct are indicated.

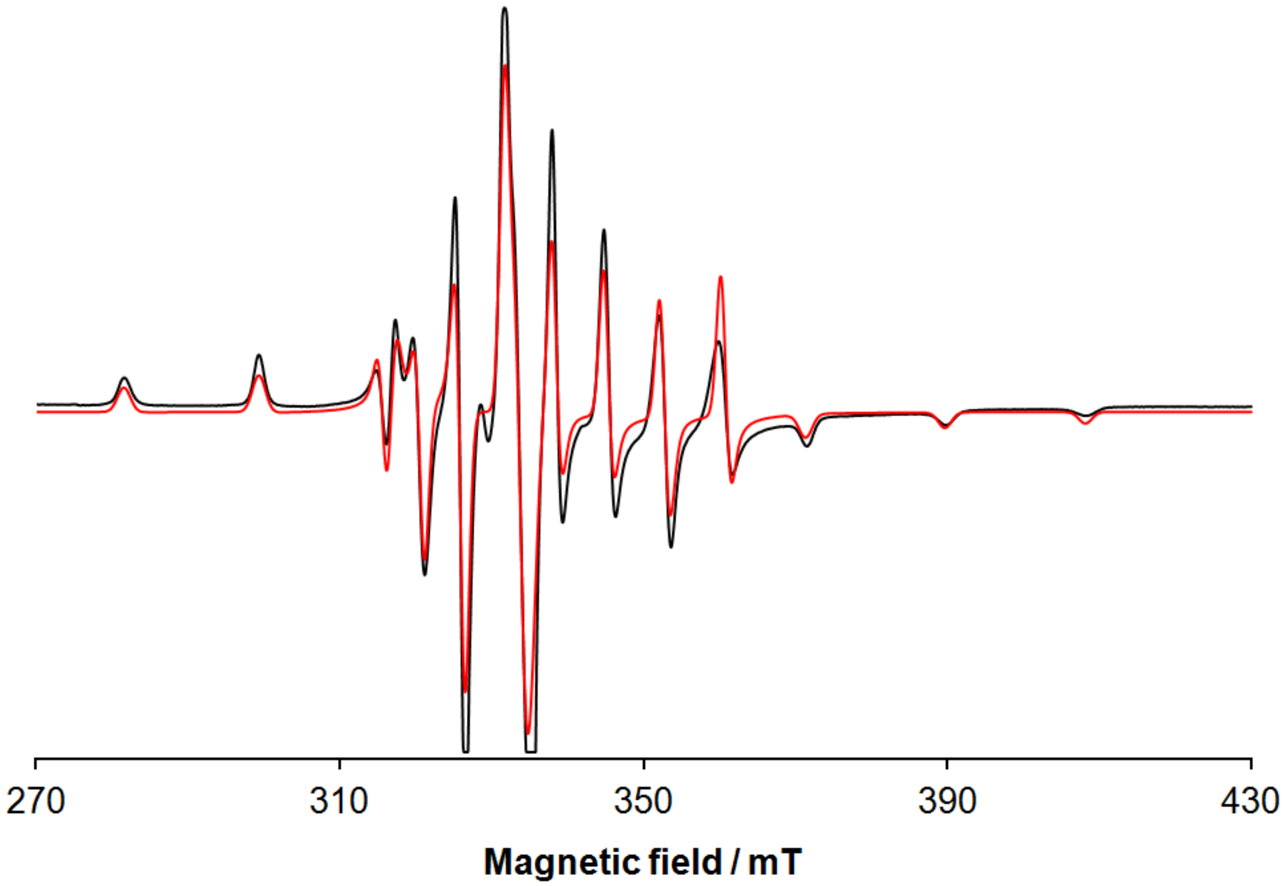

Fig. S6. Experimental (in black) and simulated (in red) spectrum of $[V^{IV}O(pic)_2(H_2O)]$. The instrumental parameters to record the spectrum were: microwave frequency 9.40433 GHz, microwave power 20.3 mW, modulation frequency 100 kHz, modulation amplitude 0.4 mT, time constant 40.96 ms, sweep time 335.5 s, resolution 2048 points. The number of scans was 1. The spin Hamiltonian parameters used for the simulation were g_x = 1.981, g_y = 1.981, g_z = 1.947, A_x = -59.0 × 10^{-4} cm^{-1}, A_y = -59.0 × 10^{-4} cm^{-1}, A_z = -164.6 × 10^{-4} cm^{-1}. The linewidth on the x, y, and z axes was 1.3, 1.3 and 1.7 mT and the Lorentzian/Gaussian ratio was 1.0. The spectrum was simulated with WinEPR SimFonia software.

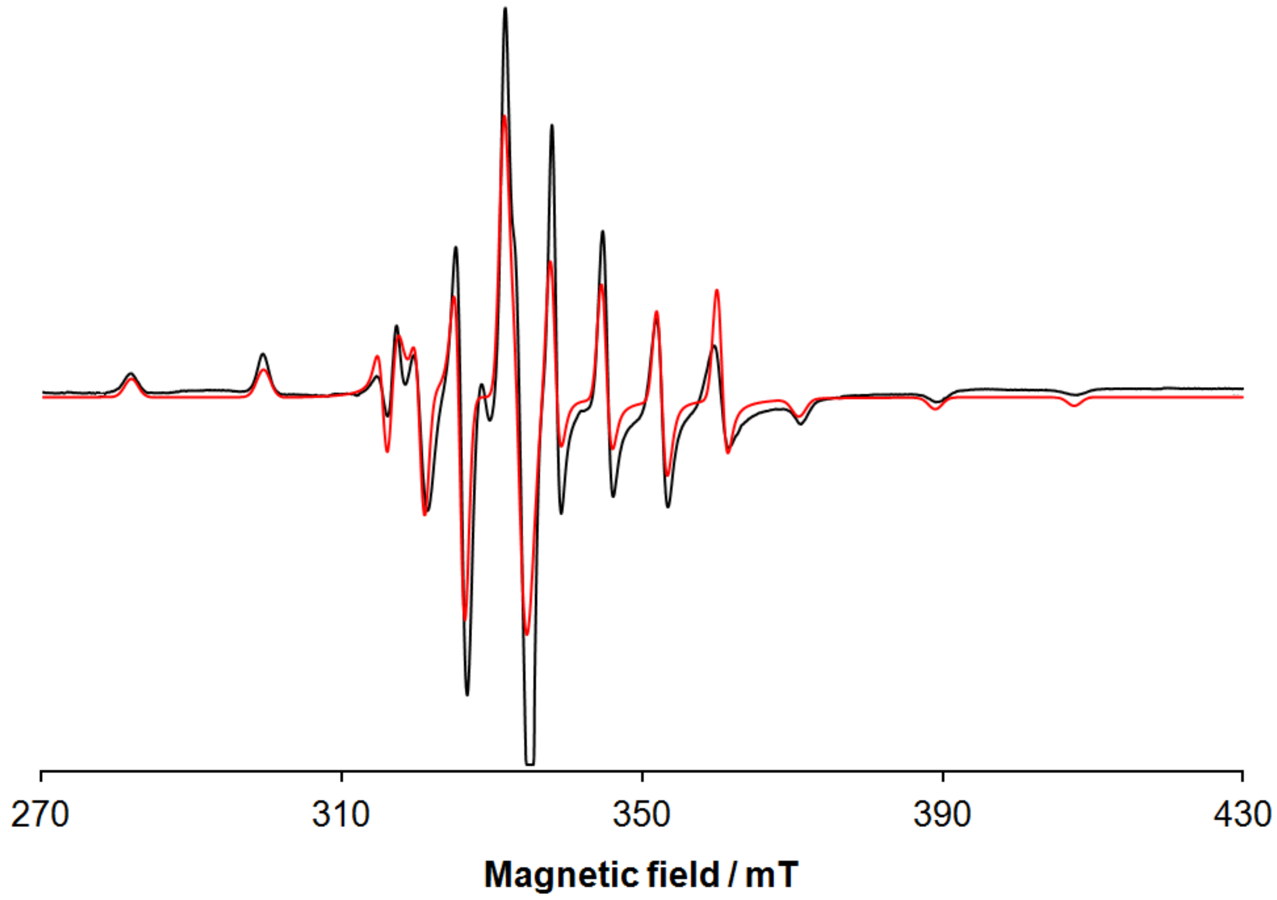

Fig. S7. Experimental and simulated spectrum of the adduct [$V^{IV}O(pic)_2$]–RNasi A with Asp/Glu binding. The instrumental parameters to record the spectrum were: microwave frequency 9.40395 GHz, microwave power 20.1 mW, modulation frequency 100 kHz, modulation amplitude 0.4 mT, time constant 40.96 ms, sweep time 335.5 s, resolution 2048 points. The number of scans was 1. The spin Hamiltonian parameters used for the simulation were g_x = 1.982, g_y = 1.982, g_z = 1.949, A_x = -58.9 × 10^{-4} cm^{-1}, A_y = -58.9 × 10^{-4} cm^{-1}, A_z = -163.0 × 10^{-4} cm^{-1}. The linewidth on the x, y, and z axes was 1.3, 1.3 and 1.8 mT and the Lorentzian/Gaussian ratio was 1.0. The spectrum was simulated with WinEPR software.

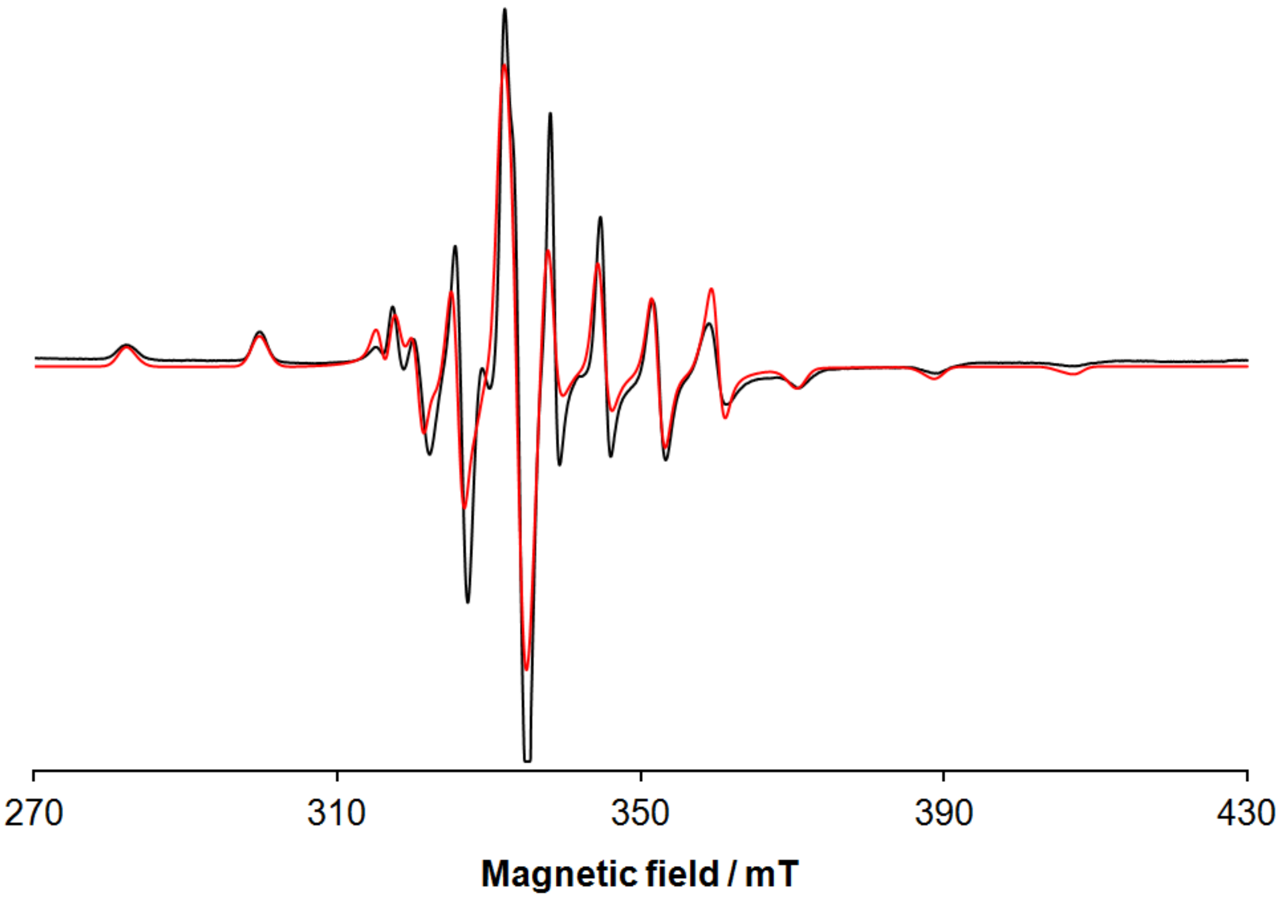

Fig. S8. Experimental and simulated spectrum of the adduct [$V^{IV}O(pic)_2$]–RNasi A with His binding. The instrumental parameters to record the spectrum were: microwave frequency 9.40306 GHz, microwave power 20.2 mW, modulation frequency 100 kHz, modulation amplitude 0.4 mT, time constant 40.96 ms, sweep time 335.5 s, resolution 2048 points. The number of scans was 1. The spectrum was simulated overlapping the signals of two species: [$V^{IV}O(pic)_2$]–RNasi A with His binding with g_x = 1.979, g_y = 1.979, g_z = 1.951, A_x = -54.0 × 10^{-4} cm^{-1}, A_y = -54.0 × 10^{-4} cm^{-1}, A_z = -159.6 × 10^{-4} cm^{-1} (linewidth on the x, y, and z axes 2.1, 2.1 and 2.4 mT) with a percent amount of 40% and [$V^{IV}O(pic)_2$]–RNasi A with Asp/Glu with g_x = 1.982, g_y = 1.982, g_z = 1.949, A_x = -58.2 × 10^{-4} cm^{-1}, A_y = -58.2 × 10^{-4} cm^{-1}, A_z = -163.0 × 10^{-4} cm^{-1} (linewidth on the x, y, and z axes 1.5, 1.5 and 2.0 mT) with a percent amount of 60%. The Lorentzian/Gaussian ratio was 1.0. The spectrum was simulated with WinEPR software.

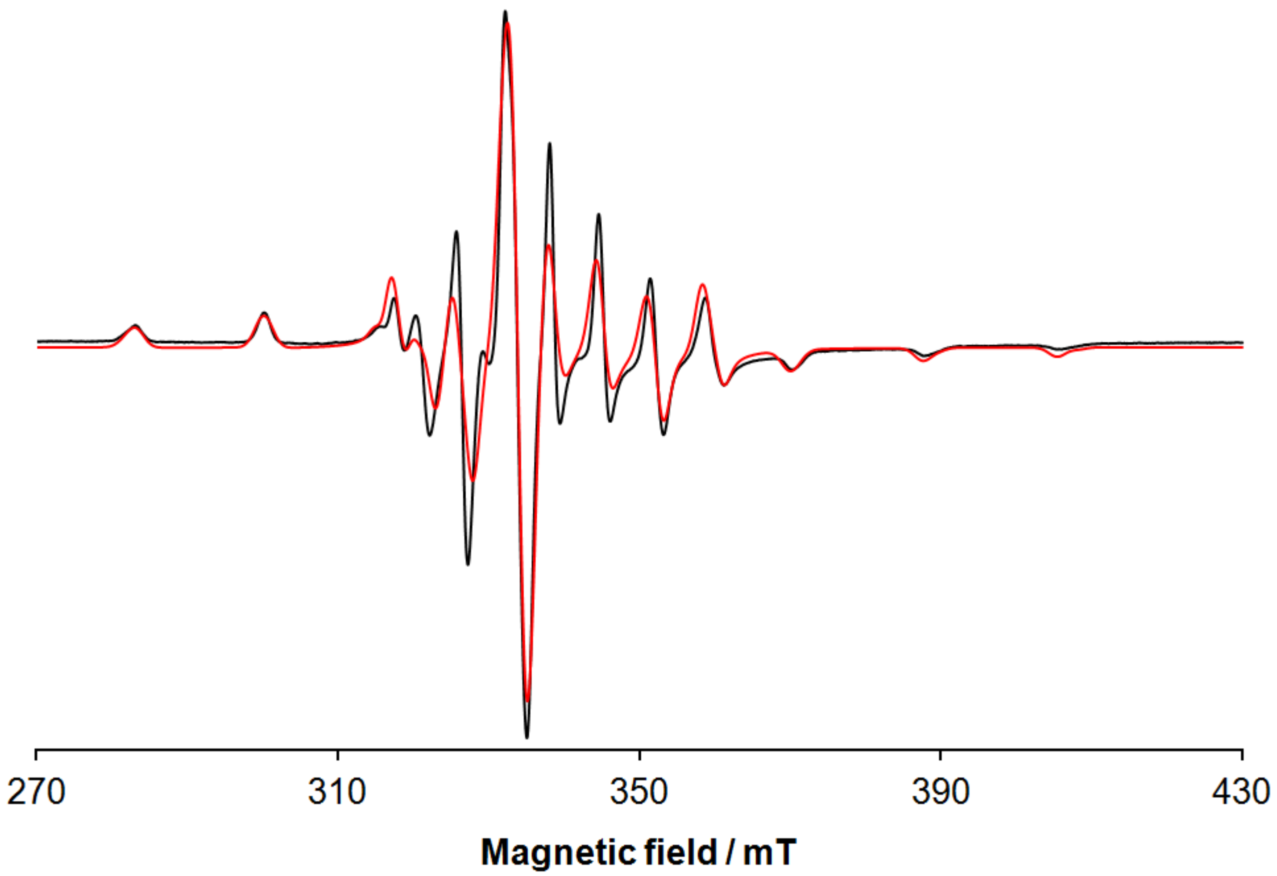

Fig. S9. Experimental and simulated spectrum of [$V^{IV}O(pic)_2(MeIm)$]. The instrumental parameters to record the spectrum were: microwave frequency 9.40358 GHz, microwave power 20.1 mW, modulation frequency 100 kHz, modulation amplitude 0.4 mT, time constant 81.92 ms, sweep time 335.5 s, resolution 4096 points. The number of scans was 1. The spectrum was simulated overlapping the signals of two species: [$V^{IV}O(pic)_2(MeIm)$] with g_x = 1.979, g_y = 1.979, g_z = 1.950, A_x = -53.8 × 10^{-4} cm^{-1}, A_y = -53.8 × 10^{-4} cm^{-1}, A_z = -159.0 × 10^{-4} cm^{-1} (linewidth on the x, y, and z axes 2.1, 2.1 and 2.2 mT) with a percent amount of 85% and [$V^{IV}O(pic)_2(H_2O)$] with g_x = 1.981, g_y = 1.981, g_z = 1.946, A_x = -59.0 × 10^{-4} cm^{-1}, A_y = -59.0 × 10^{-4} cm^{-1}, A_z = -164.6 × 10^{-4} cm^{-1} (linewidth on the x, y, and z axes 1.6, 1.6 and 2.0 mT) with a percent amount of 15%. The Lorentzian/Gaussian ratio was 1.0. The spectrum was simulated with WinEPR software.

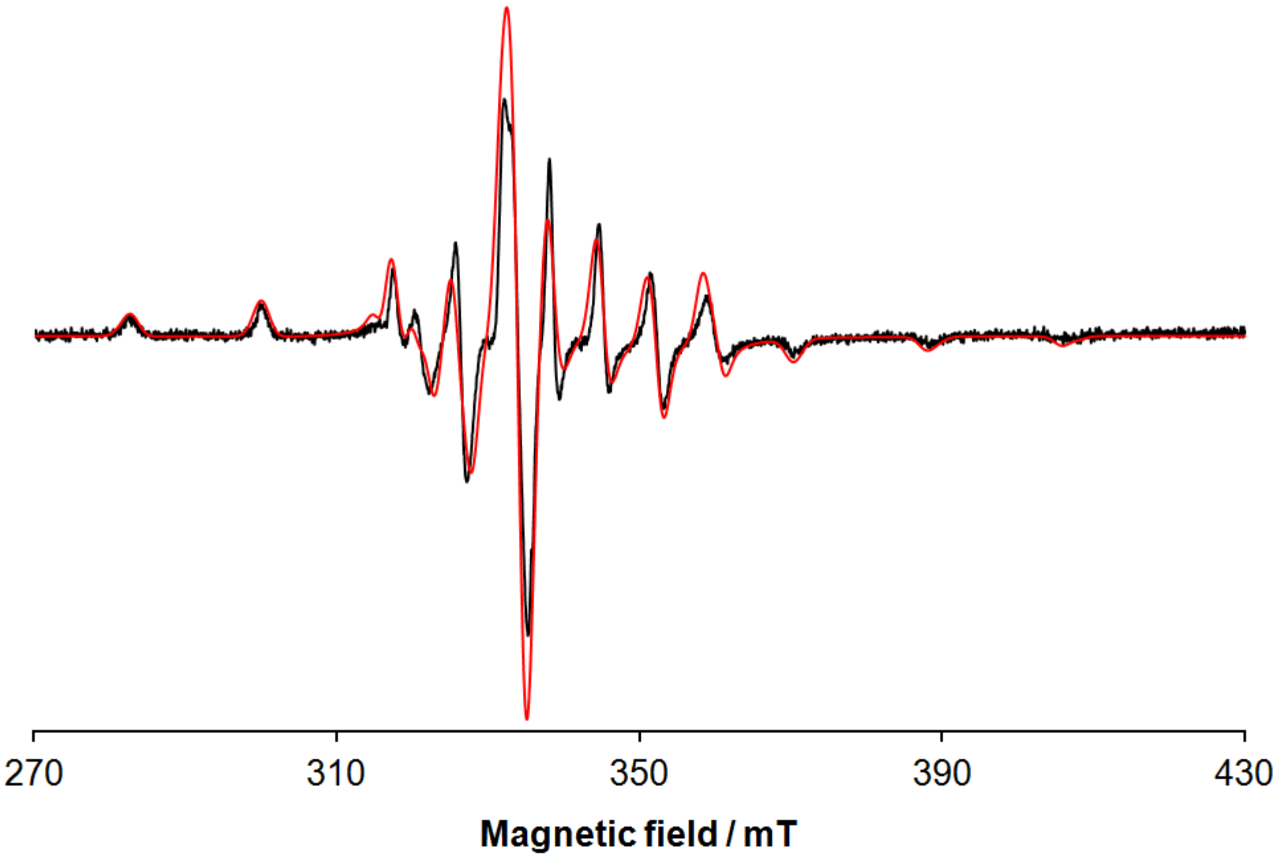

Fig. S10. Experimental and simulated spectrum of the adduct $[V^{IV}O(pic)_2]$–IgG. The instrumental parameters to record the spectrum were: microwave frequency 9.40428 GHz, microwave power 20.1 mW, modulation frequency 100 kHz, modulation amplitude 0.4 mT, time constant 81.92 ms, sweep time 335.5 s, resolution 4096 points. The number of scans was 1. The spectrum was simulated overlapping the signals of two species: $[V^{IV}O(pic)_2]$–IgG with g_x = 1.979, g_y = 1.979, g_z = 1.950, A_x = -54.1 × 10^{-4} cm^{-1}, A_y = -54.1 × 10^{-4} cm^{-1}, A_z = -159.8 × 10^{-4} cm^{-1} (linewidth on the x, y, and z axes 2.0, 2.0 and 2.0 mT) with a percent amount of 80% and $[V^{IV}O(pic)_2(H_2O)]$ with g_x = 1.981, g_y = 1.981, g_z = 1.946, A_x = -59.0 × 10^{-4} cm^{-1}, A_y = -59.0 × 10^{-4} cm^{-1}, A_z = -164.6 × 10^{-4} cm^{-1} (linewidth on the x, y, and z axes 1.6, 1.6 and 2.0 mT) with a percent amount of 20%. The Lorentzian/Gaussian ratio was 1.0. The spectrum was simulated with WinEPR software.

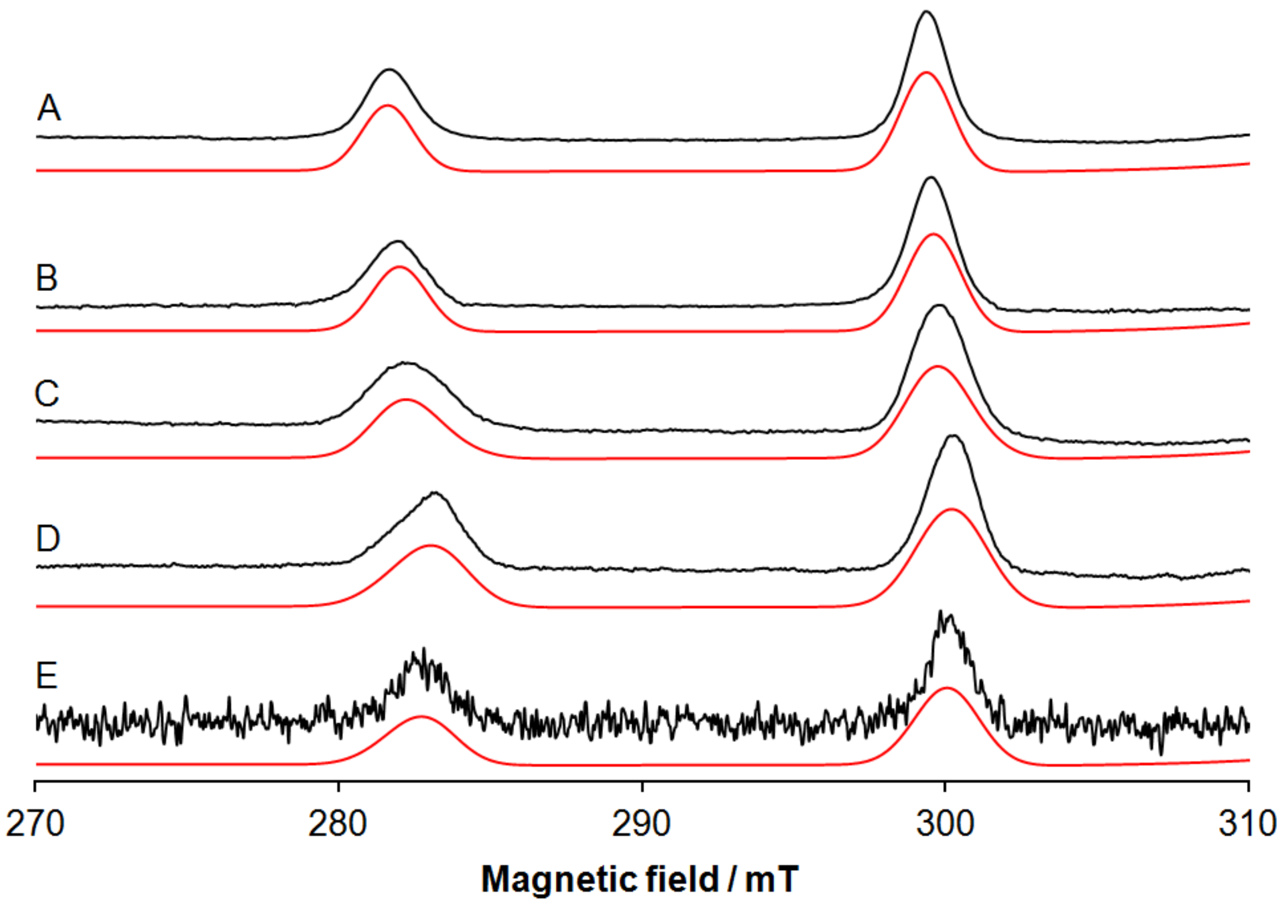

Fig. S11. Low-field region of the experimental (in black) and simulated (in red) X-band EPR spectra recorded at 120 K in an aqueous solution containing: A) $[V^{IV}O(pic)_2(H_2O)]$, V concentration 1.0 mM; B) $[V^{IV}O(pic)_2(H_2O)]$/RNase A 1/3, pH 5.4, V concentration 1.0 mM; C) $[V^{IV}O(pic)_2(H_2O)]$/RNase A 1/3, pH 7.4, V concentration 0.8 mM; D) $[V^{IV}O(pic)_2(H_2O)]$/MeIm 1/4, pH 7.4, V concentration 0.8 mM; E) $[V^{IV}O(pic)_2(H_2O)]$/IgG 1/1, pH 7.4, V concentration 0.3 mM. The number of scans was 5 for all the spectra.

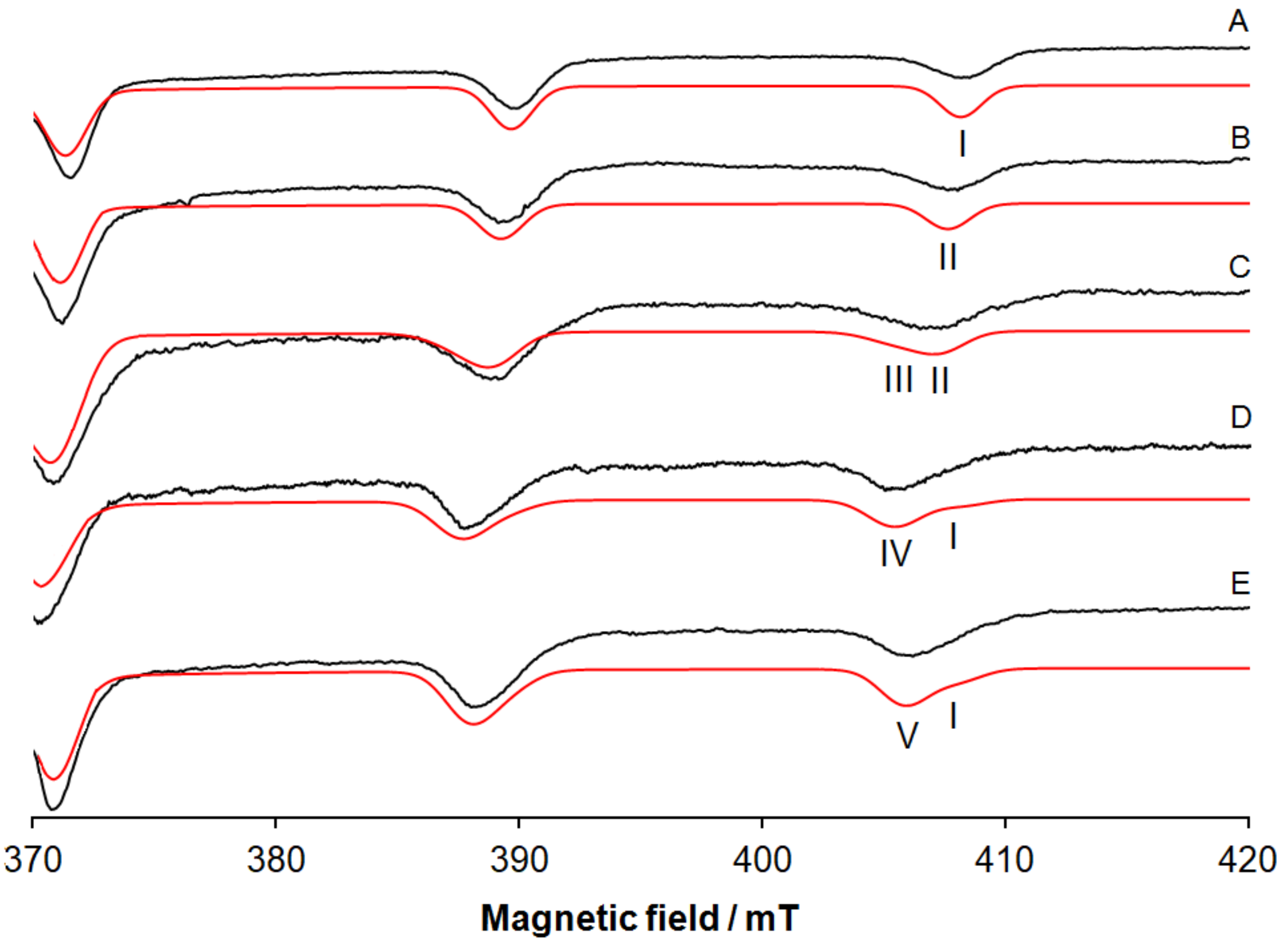

Fig. S12. High-field region of the experimental (in black) and simulated (in red) X-band EPR spectra recorded at 120 K in an aqueous solution containing: A) $[V^{IV}O(pic)_2(H_2O)]$, V concentration 1.0 mM; B) $[V^{IV}O(pic)_2(H_2O)]$/RNase A 1/3, pH 5.4, V concentration 1.0 mM; C) $[V^{IV}O(pic)_2(H_2O)]$/RNase A 1/3, pH 7.4, V concentration 0.8 mM; D) $[V^{IV}O(pic)_2(H_2O)]$/MeIm 1/4, pH 7.4, V concentration 0.8 mM; E) $[V^{IV}O(pic)_2(H_2O)]$/IgG 1/1, pH 7.4, V concentration 0.3 mM. **I** indicates the M_I = 7/2 resonances of $[V^{IV}O(pic)_2(H_2O)]$, **II** of the $[V^{IV}O(pic)_2]$–RNase A adduct with Asp/Glu-COO^- coordination, **III** of the $[V^{IV}O(pic)_2]$–RNase A adduct with His-N coordination, **IV** of the $[V^{IV}O(pic)_2(MeIm)]$ complex and **V** of the $[V^{IV}O(pic)_2]$–IgG adduct with His-N coordination, using the same symbols as in Fig. 2 of the main text. The number of scans was 5 for the traces a-d and 10 for the trace e.

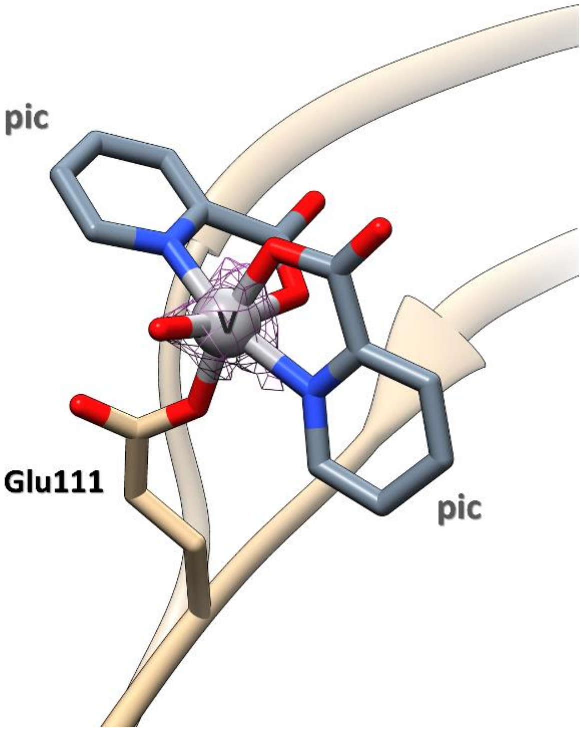

Fig. S13. Anomalous difference electron density map (in purple at 3.0σ level) in correspondence of V atom, close to the side chain of Glu111.

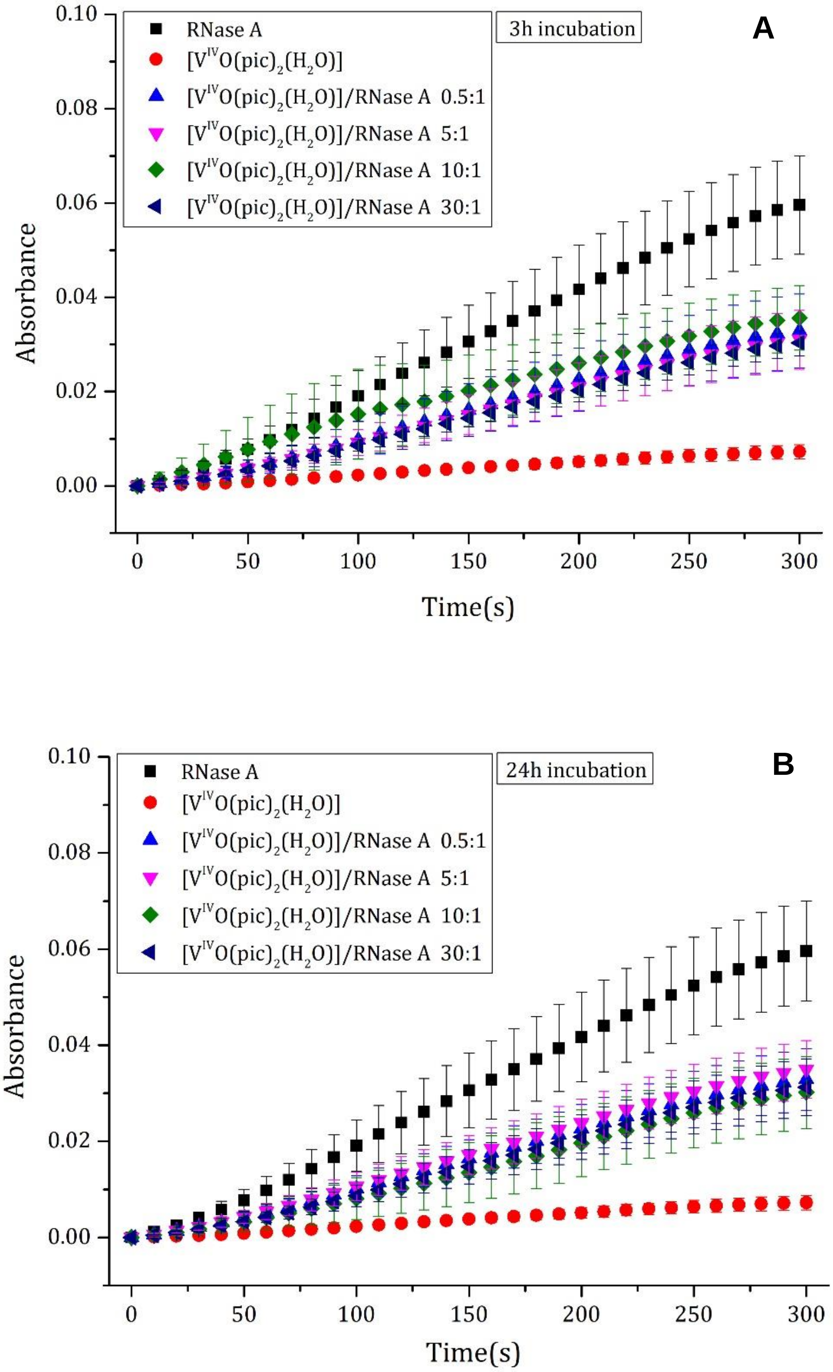

Fig. S14. Hydrolysis of yeast RNA (evaluated by measuring the variation of absorbance at 300 nm as function of time upon addition of the protein to the yeast RNA sample) by native RNase A (black squares) and RNase A in the presence of different concentrations of $[V^{IV}O(pic)_2(H_2O)]$ (colored triangles and circles). RNase A was incubated for 3 h (A) and 24 h (B).

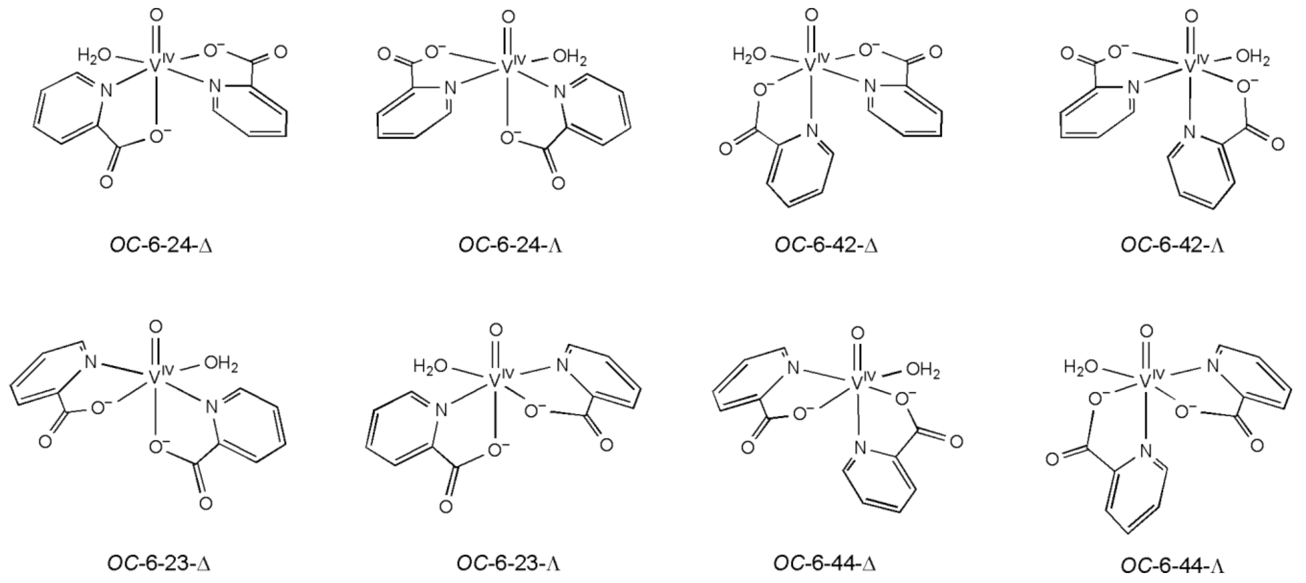

Scheme S1. Possible isomers of [$V^{IV}O(pic)_2(H_2O)$]. The more stable isomers are *OC*-6-23-Δ/Λ and *OC*-6-24-Δ/Λ with the two nitrogen atoms in the equatorial plane of the $V^{IV}O^{2+}$ ion.
